# Exploring immune response toward transplanted human kidney tissues assembled from organoid building blocks

**DOI:** 10.1016/j.isci.2024.110957

**Published:** 2024-09-13

**Authors:** Thiago J. Borges, Yoshikazu Ganchiku, Jeffrey O. Aceves, Ronald van Gaal, Sebastien G.M. Uzel, Ivy A. Rosales, Jonathan E. Rubins, Kenichi Kobayashi, Ken Hiratsuka, Murat Tekguc, Guilherme T. Ribas, Karina Lima, Rodrigo B. Gassen, Ryuji Morizane, Jennifer A. Lewis, Leonardo V. Riella

**Affiliations:** 1 Center for Transplantation Sciences, Massachusetts General Hospital, Harvard Medical School, Boston, MA, United states; 2 Paulson School of Engineering and Applied Sciences, Harvard University, Cambridge, MA, United states; 3 Wyss Institute for Biologically Inspired Engineering, Harvard University, Boston, MA, United states; 4 Department of Pathology, Massachusetts General Hospital and Harvard Medical School, Boston, MA, United states; 5 Nephrology Division, Department of Medicine, Massachusetts General Hospital, Harvard Medical School, Boston, MA, United states; 6 Harvard Stem Cell Institute (HSCI), Cambridge, MA, United states

**Keywords:** Health sciences, Immunology, Bioengineering, Tissue engineering

## Abstract

The increasing scarcity of organs and the significant morbidity linked to dialysis require the development of engineered kidney tissues from human-induced pluripotent stem cells. Integrative approaches that synergize scalable kidney organoid differentiation, tissue biomanufacturing, and comprehensive assessment of their immune response and host integration are essential to accomplish this. Here, we create engineered human kidney tissues composed of organoid building blocks (OBBs) and transplant them into mice reconstituted with allogeneic human immune cells. Tissue-infiltrating human immune cells are composed of effector T cells and innate cells. This immune infiltration leads to kidney tissue injury characterized by reduced microvasculature, enhanced kidney cell apoptosis, and an inflammatory gene signature comparable to kidney organ transplant rejection in humans. Upon treatment with the immunosuppressive agent rapamycin, the induced immune response is greatly suppressed. Our model is a translational platform to study engineered kidney tissue immunogenicity and develop therapeutic targets for kidney rejection.

## Highlights

• Like human transplants, engineered iPSCs-derived kidney tissues face immune rejection
• Immune infiltration causes microvascular injury and cell death in humanized mouse model
• Engineered kidney tissues mimic rejection and aid in testing immunosuppressive drugs
• Rapamycin prevents rejection and preserves the integrity of transplanted kidney tissues

## Graphical abstract

**Figure:**
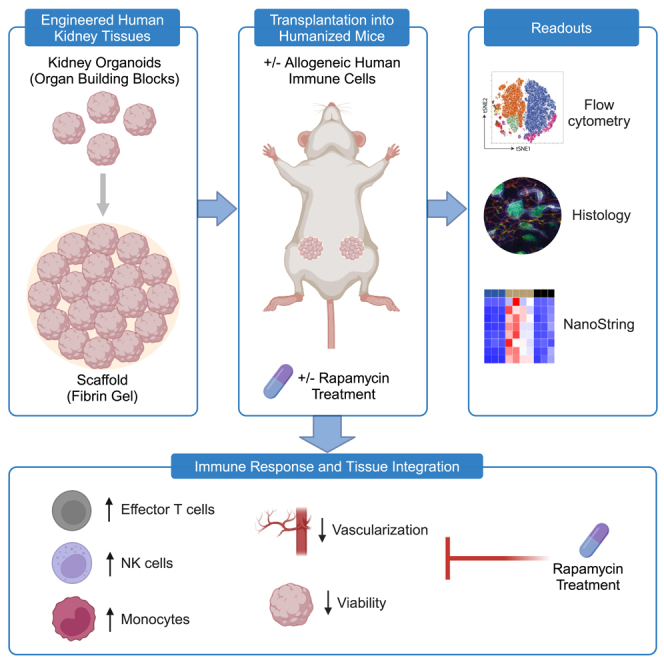

## Introduction

The kidneys are vital organs that maintain homeostasis by controlling fluid and electrolyte balance, removing waste products, and regulating blood pressure. In the US alone, roughly 25 million people have chronic kidney disease (CKD).^1^^,^^2^ Many of these patients will progress to end-stage renal disease (ESRD), for which kidney transplantation is the best treatment. However, due to the lack of transplantable organs, patients typically require dialysis. Although dialysis is capable of helping with fluid and electrolyte balance,^3^ it is associated with substantial morbidity and mortality primarily due to cardiovascular disease.^3^^,^^4^ The five-year survival rate of people on hemodialysis is less than 50%— worse than some types of cancer.^1^ By contrast, kidney transplantation increases patient survival, reduces costs, and improves their quality of life.^5^^,^^6^ In the US alone, more than 650,000 people are on dialysis and ∼95,000 people await kidney transplants with over 3,000 new patients added monthly to the wait list (http://optn.transplant.hrsa.gov). Given the growing shortage of organs and the substantial morbidity associated with dialysis, there is a need to develop alternative sources of kidney tissue that can restore normal kidney function.

We postulate that biomanufacturing functional kidney tissues and, ultimately, whole organs may offer an important solution to this growing problem. Toward this goal, engineered human kidney tissues must be scalably produced and successfully integrated with the host upon transplantation. Recently, differentiation protocols have been developed to generate “mini-kidneys” from human-induced pluripotent stem cells (hiPSCs).^7^^,^^8^^,^^9^^,^^10^ These organoids exhibit remarkable tissue microarchitectures with high cellular density akin to their *in vivo* counterparts. Integrative approaches that combine scalable organoid differentiation with tissue biomanufacturing as well as the evaluation of immune responses and host integration are needed to begin to close the gap from organoid building blocks (OBBs) to therapeutic organs.^11^

Here, we created engineered human kidney tissues assembled from kidney OBBs and assessed their *in vivo* vascular integration, maturation, and elicited immune responses in a humanized mouse model. Kidney organoids were differentiated from the hiPSCs, suspended in a fibrinogen solution, compacted into a dense tissue matrix, and then patterned into thin tissue discs (3 mm in diameter, 1 mm in thickness, with a total volume of ∼10 mm^3^). Their final composition is akin to the living tissue matrices used for a bioprinting method known as sacrificial writing into functional tissue.^11^^,^^12^ Induced pluripotent stem (iPS) cells were human leukocyte antigen (HLA) typed, and kidney tissues were transplanted into the fat pad of NOD-*scid* IL2Rgamma^null^ (NSG) mice reconstituted with allogeneic human immune cells. Analysis of the infiltrating human immune cells within the kidney tissues revealed the presence of activated CD4^+^ and CD8^+^ T cells as well as innate cells, such as monocytes and natural killer cells. This correlated with enhanced cytotoxic activity, and a distinctive inflammatory gene signature within the transplanted kidney tissues. Subsequently, we observed transplant rejection features characterized by an increase in kidney apoptotic cells and reduced tissue vascularization and maturation markers. However, treatment with the immunosuppressive agent, rapamycin, effectively inhibited the human immune response and prevented kidney tissue injury. Our integrated platform enables the investigation of HLA-incompatible immune responses to human-engineered human organoid-based tissues, as well as the efficacy and nephrotoxicity of new therapeutical agents.

## Results

### Engineering kidney tissues from OBBs

**Figure 1:**
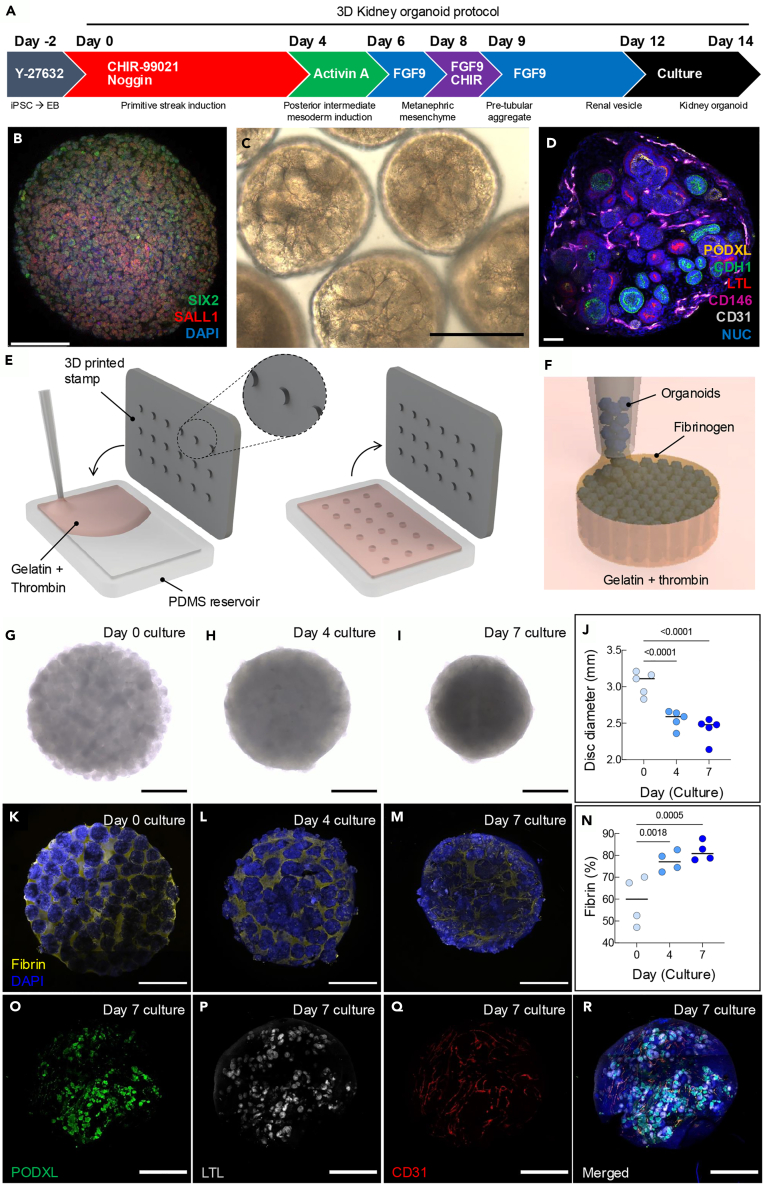
Generation and characterization of human kidney tissues assembled from organoid building blocks (A) 3D differentiation protocol used to generate kidney organoids from hiPSCs. (B) Immunostaining on day 9 of differentiation confirming metanephric mesenchymal markers SIX homeobox 2 (SIX2, green) and spalt-like transcription factor 1 (SALL1, red); scale bar, 100 μm. (C and D) Confocal images of kidney organoids differentiated and cultured in spinner flasks (day 14), confirming expression of podocyte (PODXL, green), proximal tubule (LTL, red), distal tubule (CDH1, green), and endothelial (CD31, gray) cell populations; scale bars, 40 μm. (E and F) Schematic illustrations of kidney tissue disc fabrication method, in which a 3D printed stamp is molded into a PDMS reservoir that contains a gelatin and thrombin mixture to pattern cylindrical features of diameter = 3 mm and height = 1 mm. Kidney tissue discs are produced by filling these molded features with a densely cellular assembly of kidney organoids suspended in a fibrinogen solution, which rapidly polymerizes into fibrin via diffusion of thrombin from the surrounding gelatin matrix. (G–I) Optical images of the kidney tissue discs cultured at days 0, 4, and 7 on an orbital shaker; scale bars, 1 mm. (J) Plot of kidney tissue disc diameter as a function of culture time, which reflects organoid fusion into a contiguous tissue, two-way ANOVA, ∗∗∗ = *p* < 0.001. (K–M) Confocal images showing the reduction in fibrin (yellow) content as a function of culture time (days 0, 4, and 7) for these kidney tissue discs; scale bars, 1 mm. (N–R) (N) Corresponding plot of fibrin (yellow) content as a function of culture time for these tissues, which reflects the measured area fraction of fibrin determined by image analysis, two-way ANOVA, ∗∗ = *p* < 0.01, ∗∗∗ = *p* < 0.001. After 7 days of static culture and tissue fusion, these kidney organoid-based tissues still contained (O) podocyte (PODXL, green), (P) proximal tubule (LTL, gray), and (Q) endothelial (CD31, red) cells that self-organize into (R) glomerular, tubular, and microvascular architectures, respectively; scale bars, 1 mm.

Human kidney tissues are generated by creating kidney organoids differentiated from hiPSCs by following our previously published protocol of directed differentiation,^7^^,^^10^ with modifications for stirred bioreactors or spinner flasks that enable 3D differentiation (Figure 1A). This protocol involves differentiation into metanephric mesenchyme, as confirmed by SIX2 and SALL1 immunostaining on day 9 of differentiation (Figure 1B). The resulting kidney organoids exhibit glomerular and tubular features under bright-field imaging (Figure 1C), staining positive for podocyte (podocalyxin-like protein 1, PODXL), proximal tubule (lotus tetragonolobus lectin, LTL), distal tubule (cadherin 1, CDH1), and endothelial (platelet endothelial cell adhesion molecule, PECAM1/CD31) markers (Figure 1D). The kidney organoids are then suspended in a fibrinogen-based (10 mg/mL) extracellular matrix solution and compacted via centrifugation to form a densely cellular tissue matrix, which is cast into a mold containing gelatin and thrombin. The mold is pre-patterned using a 3D printed stamp to produce a periodic array of 18 cylindrical features (diameter = 3 mm and height = 1 mm, Figure 1E). The gelatin and thrombin mold is initially cast at 37°C, and features are produced by adding the stamp, cooling to 4°C, and removing the stamp, leaving the desired cavities. Upon casting the organoid-fibrinogen solution into these cylindrical molds, the thrombin diffuses from the surrounding extracellular gelatin matrix to promote its rapid polymerization to fibrin, which initially binds the organoids together before their fusion (Figure 1F). The kidney tissue discs are cultured for 7 days to promote organoid fusion prior to implantation. This process leads to a pronounced reduction in tissue disc diameter (Figures 1G–1J) as well as a diminishing presence of fibrin over this period (Figures 1K–1N). Importantly, these kidney tissue discs continue to exhibit robust expression of podocytes and proximal tubular markers, and early stages of microvessel formation, as reflected by CD31 expression (Figures 1O–1R).

### Immune response to the transplanted engineered kidney tissues

**Figure 2:**
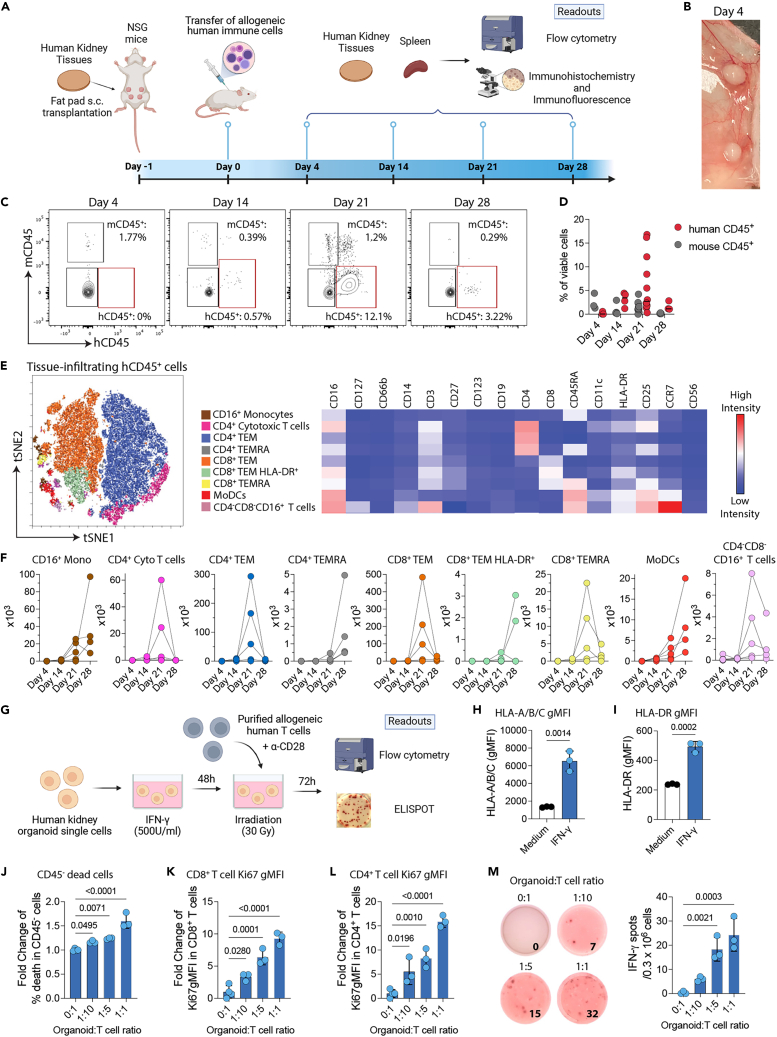
Immune response to transplanted kidney organoid-based tissues (A) Kidney tissues were subcutaneously (s.c.) transplanted into the fat pad of NSG mice reconstituted with or without allogeneic human immune cells (the source is freshly isolated PBMCs). The kidney tissues were recovered and analyzed by histology and flow cytometry on days 4, 14, 21, and 28 after allogeneic human immune cells were transferred. (B) Image of the kidney tissues harvested on day 4. (C and D) (C) Representative contour plots (gated on viable singlets) and (D) percentages of mouse and human kidney tissue-infiltrating CD45^+^ leukocytes over time. (E) The viSNE plot and heatmap represent the expression of immune markers in nine immune cell meta-clusters identified by FlowSOM. (F) Absolute numbers of tissue-infiltrating human CD16^+^ monocytes, CD4^+^ cytotoxic T cells, CD4^+^ T effector memory (TEM), CD4^+^ T effector memory CD45RA^+^ (TEMRA), CD8^+^ TEM, CD8^+^HLA-DR^+^TEM, monocyte-derived dendritic cells (MoDCs), and CD4^−^CD8^−^CD16^+^ T cells on days 4, 14, 21, and 28 after the transfer of allogeneic human immune cells. Data are representative of two pooled experiments (*n* = 3–5 tissues/group/time point). (G) Schematic illustration of the experimental setup. Single cells from kidney organoids were stimulated with 500 U/ml of recombinant human IFN-γ for 2 days, washed twice, irradiated, and co-cultured with purified allogeneic T cells for 3 days until the readout assay was performed. (H and I) (H) Human leukocyte antigen A-A/B/C (HLA-A/B/C) or (I) HLA-DR expression quantified by flow cytometry analysis in kidney organoids cultured with or without (medium) 500 U/mL of recombinant human IFN-γ for 2 days. gMFI, geometric mean fluorescence intensity. Statistical analyses are done by unpaired t tests. (J–L) Fold change of the percentages of (J) dead cells within the CD45^−^ population when kidney cells were cultured at different ratios with purified allogeneic T cells. Ki67 expression by (K) CD8^+^ or (L) CD4^+^ T cells when co-cultured with different ratios of kidney cells. gMFI, geometric mean fluorescence intensity. (J–L) Data are expressed as a fold change compared to 0:1 organoid:T cell ratio. (M) IFN-γ production was measured using Enzyme-linked immunosorbent spot (ELISPOT) by purified allogeneic T cells when co-cultured with different ratios of kidney cells (number of spots per 0.3 million T cells ±SD in triplicates). (J and M) Statistics are performed by one-way ANOVA with Tukey’s multiple comparisons test. Data are representative of two independent experiments.

To investigate the immune responses triggered by the kidney organoid-based tissue, we subcutaneously transplanted kidney organoid tissue discs (∼10 mm^3^) into the fat pad of the immunocompromised NSG mice. On the following day, the transplanted NSG mice were reconstituted with allogeneic human immune cells (peripheral blood mononuclear cells, PBMCs) isolated from a healthy volunteer or PBS 1x (controls). We then evaluated the immune response in the kidney discs and spleens over time (Figure 2A). Prior to transplantation, the iPSCs and immune cells were HLA typed, and four HLA mismatches on A, B, and DR loci (A1, B2, and DR1) were identified at the antigen level (Table S1). To improve the precision of the alloimmune risk assessment, we also characterized the donor-recipient HLA mismatch at the molecular level.^13^ Our data revealed 41 eplet mismatches in HLA class I and 23 in class II.

At the tissue scale, we observed host integration and vascularization of transplanted kidney tissues harvested on days 4, 14, 21, and 28 after transferring allogeneic human immune cells (Figures 2B and S1A). There was no difference between the gross weights of discs harvested from NSG mice with or without an allogeneic human immune system (Figure S1B). To control for the presence of off-target cells, we stained the harvested kidney tissues for the pluripotency markers TRA-1-60, Oct3/4, and SSEA-4^14^ and analyzed their expression by flow cytometry, using hiPSCs as positive controls. While almost 100% of hiPSCs were positive, none of the harvested kidney cells expressed any of these three markers (Figures S1C–S1E). This is in accordance with our previously published study demonstrating minimum off-target cells by single-nucleus RNA sequencing.^15^ Hence, these kidney tissues are composed solely of differentiated cells with no potential to generate off-target cell types.

We next evaluated the infiltration of human and mouse CD45^+^ leukocytes over time. Our data demonstrated that human immune cell infiltration peaked within these kidney tissues at day 21 post-PBMC injection (Figures 2C and 2D). T cells are the major drivers of kidney transplant rejection.^16^ To characterize the subsets of the human immune cells infiltrating the transplanted tissues, we performed an unsupervised hierarchical clustering by employing in-depth immune phenotyping with an 18-marker flow cytometry panel. We identified 9 tissue-infiltrating human immune cell populations, with the majority of cells composed of T cells (Figure 2E). Within the T cell populations, we found that CD4^+^ cytotoxic (CD16^+^) T cells, CD4^+^ and CD8^+^ T effector memory (TEM), and CD8^+^ T effector memory that express CD45RA (TEMRA) peaked on day 21 after the transfer of allogeneic human immune cells (Figure 2F). From the innate compartment, we identified CD16^+^ monocytes and monocyte-derived dendritic cells (MoDCs) populations that increased over time, peaking at day 28 (Figure 2F). Similar to the effector T cells, the absolute number of CD4^−^CD8^−^CD16^+^ T cells increased on day 21 (Figure 2F). The increased infiltration of CD8^+^ and CD4^+^ mononuclear cells over time was confirmed by immunohistochemistry (Figures S2A and S2B). Importantly, all observed immune subsets were associated with an effector/activated phenotype.

We then investigated whether differentiated kidney cells are recognized by total allogeneic purified T cells *in vitro*. We initially observed that kidney cells isolated from organoids upregulate the expression of HLA class I (HLA-A, B, and C, Figures 2G and 2H) and II (HLA-DR, Figure 2I) when stimulated with recombinant human interferon (IFN)-γ. We carried out two different assays to assess T cell-mediated activation and cytotoxicity in our model. Allogeneic human purified T cells (responders) are co-cultured with different numbers of IFN-γ-stimulated and irradiated kidney cells (stimulators, Figure 2G). There is a proportional increase in cell death within the CD45^−^ population (non-immune cells) when kidney cells are exposed to allogeneic human purified T cells (Figure 2J). Both CD8^+^ and CD4^+^ T cells exhibit an increasing fold change in the expression of the proliferation marker Ki67 (Figures 2K and 2L) and in the production of the effector cytokine IFN-γ when evaluated by Enzyme-linked immunosorbent spot (ELISPOT) (Figure 2M). Our data indicate that allogeneic human T cells both recognize and proliferate in response to differentiated kidney cells, leading to cell death. Overall, our findings indicate that the transplantation of human kidney tissues assembled from OBBs in the presence of allogeneic human immune cells can generate effector innate and adaptive immune responses against the allo-kidney tissue.

### *In vivo* expression of kidney markers by transplanted tissues

**Figure 3:**
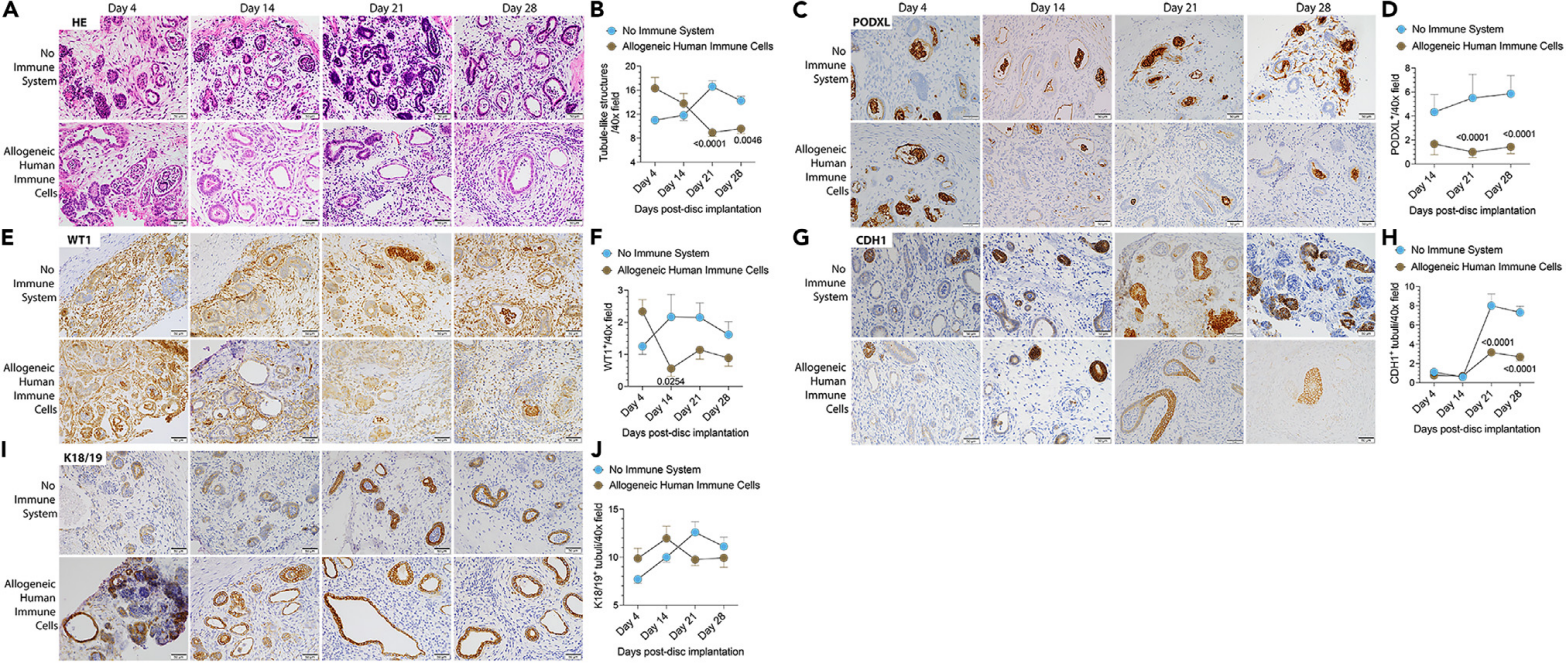
Transplanted kidney tissues express markers of different kidney cell subsets Kidney tissues were transplanted in NSG mice reconstituted with or without allogeneic human immune cells. (A and B) Representative kidney tissue H&E staining and quantification of tubuli-like structures over time. (C–J) Immunohistochemical expression analyses quantification of (C and D) PODXL (podocytes), (E and F) WT-1 (podocytes), (G and H) CDH1 (distal tubules), and (I and J) K18/19 (distal tubules) positive structures on days 4, 14, 21, and 28 after the transfer of allogeneic human immune cells. Data are representative of three independent experiments. Statistical analyses are done by two-way ANOVA followed by Šídák’s multiple comparison tests (*n* = 4–28 tissues/group/time point). Scale bars, 50 μm.

The *in vivo* viability of kidney OBBs is an important attribute for their use in creating engineered tissues and, ultimately, transplantable organs.^17^ Hence, we evaluated the nephron epithelia of our transplanted tissues in the absence or presence of allogeneic human immune cells. Histologically, we found an increase in tubule-like structures over time, which is significantly reduced in the presence of an allogeneic human immune system (Figures 3A and 3B). Moreover, increasing interstitial inflammation is observed on days 21 and 28 after the transfer of allogeneic human immune cells (Figure 3A). Immunohistochemical studies using podocalyxin (PODXL, podocytes), Wilms’ tumor-1 (WT-1, podocytes), E-cadherin (CDH1, distal tubule), and urinary keratin K18/19 (K18/19, distal tubule) demonstrated that our transplanted kidney tissue discs exhibited glomerular (PODXL^+^, Figures 3C and 3D; WT-1^+^, Figures 3E and 3F) and tubular (CDH1^+^, Figures 3G and 3H) structures that were maintained or increased over time in the absence of allogeneic human immune cells. However, in the presence of allogeneic human immune cells, these structures were significantly degraded except for the K18/19 marker (Figures 3A–3J). Our data clearly show that transplanted kidney tissues remain intact *in vivo* after implantation in NSG mice, while the introduction of allogeneic human immune cells leads to injury of glomerular and tubular structures.

### Allogeneic human immune cells induce engineered kidney tissue rejection

**Figure 4:**
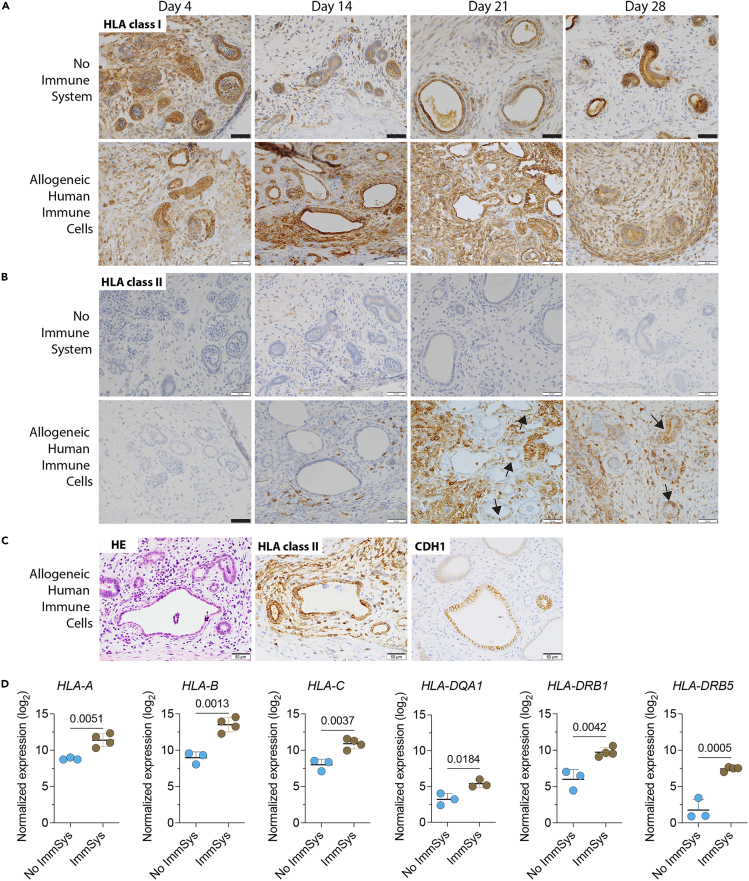
Kidney organoid-based tissues express HLA class I and II *in vivo* (A–B) Kidney tissues are transplanted into NSG mice reconstituted with or without allogeneic human immune cells, harvested, and then analyzed on days 4, 14, 21, and 28. Immunostaining of kidney tissues (A) HLA class I (HLA-A, B, and C) and (B) HLA class II (HLA-DR) expression over time. Data are representative of two independent experiments. Arrows indicate tubules expressing HLA-DR. Scale bars, 50 mm. (C) Consecutive kidney tissue sections were stained for H&E, HLA-DR, and the tubular marker CDH1 on day 21. Scale bars, 50 mm. (D) Log_2_ normalized gene expression of disc *HLA-A*, *HLA-B*, *HLA-C*, *HLA-DQA1*, *HLA-DRB1*, and *HLA-DRB5* transcripts from NSG mice with no immune system (No ImmSyst, *n* = 3) and reconstituted with allogeneic human immune cells (ImmSyst, *n* = 4) by NanoString analysis. Statistical analyses are done by unpaired t tests.

During transplantation, the recipient’s immune system recognizes donor HLA molecules as foreign and initiates a rejection response.^16^ To evaluate kidney tissue immunogenicity following *in vivo* transplantation, we stained the harvested tissues for HLA class I (HLA-A, B, and C) and II (HLA-DR). We found that the kidney tissues expressed HLA I in the absence of allogeneic human immune cells over time (Figure 4A). By contrast, HLA II was not detected in the group without immune cells (Figure 4B). In the presence of allogeneic human immune cells, HLA class I immunostaining increased (Figure 4A), including expression by interstitial infiltrating mononuclear cells. Furthermore, in NSG mice reconstituted with allogeneic human immune cells, harvested kidney tissues exhibited increased interstitial HLA-DR^+^ immune cells, reaching a peak on day 21 (Figure 4B). These data confirm our flow cytometry findings regarding the infiltration of both antigen-presenting cells and HLA-DR^+^ T cells within these transplanted kidney tissues (Figures 2D and 2E). While HLA class II molecules are primarily expressed by antigen-presenting cells, kidney tubules can upregulate HLA class II in the setting of injury.^18^ Indeed, we observe tubular expression of HLA-DR on days 21 and 28 after the transfer of allogeneic human immune cells (Figure 4B, arrow). This is confirmed by staining of consecutive kidney tissue sections for H&E, HLA-DR, and the tubular marker CDH1, which reveals that HLA-DR^+^ and CDH1^+^ tubular cells are co-localized (Figures 4C and S3). Using transcriptomics, we evaluated the discs on day 21 by NanoString and confirmed an enhanced presence of HLA class I (HLA-A, B, C) and HLA class II (HLA-DQA1, DRB1, and DRB5) transcripts in tissues transplanted in mice reconstituted with allogeneic human immune cells compared to controls (Figure 4D).

**Figure 5:**
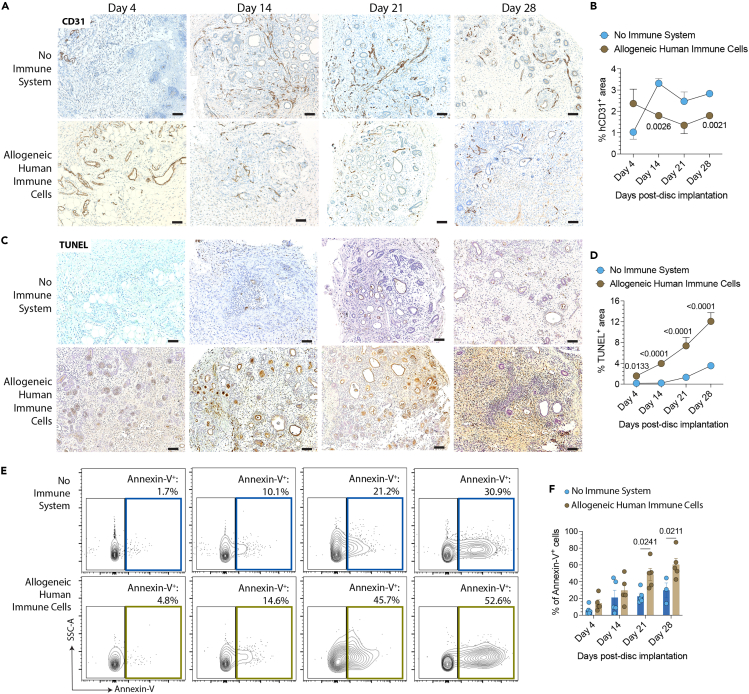
Transplanted kidney tissues are rejected by allogeneic human immune cells *in vivo* Kidney tissues are transplanted into NSG mice reconstituted with or without allogeneic human immune cells, harvested, and then analyzed on days 4, 14, 21, and 28. (A–D) Immunostaining and quantification of transplanted kidney tissue (A and B) CD31 (endothelial cells) and (C and D) TUNEL (apoptosis) expression over time. Scale bars, 100 mm. Data are representative of three independent experiments. (E and F) (E) Representative contour plots and (F) quantification of Annexin-V^+^ (apoptotic) cells by flow cytometry on kidney tissues harvested over time. Statistical analyses are done by two-way ANOVA followed by Šídák’s multiple comparison tests (*n* = 3–5 tissues/group/time point).

To assess host integration and vascularization in the absence or presence of allogeneic human immune cells, we stained and quantified the endothelial marker CD31 in these transplanted kidney tissues. We find that the number of CD31^+^ vessels increased on day 14 post-transplantation and remained stable over time in the absence of immune cells by immunohistochemistry (Figures 5A and 5B). By contrast, the CD31^+^ vessels decreased in tissues exposed to allogeneic human immune cells (Figures 5A and 5B), suggesting enhanced injury to the vascular endothelium. Confocal imaging confirmed the decrease of CD31^+^ cells and maturation markers in kidney tissues harvested from NSG mice reconstituted with allogeneic human immune cells (Figures S4A and S4B). Next, we used the TUNEL assay to detect DNA-strand breaks, which are characteristic of apoptotic cells.^19^ Apoptosis of cells within the transplanted kidney tissues increased markedly in NSG mice reconstituted with allogeneic human immune cells (Figures 5C and 5D). We further confirmed these findings by staining the harvested tissues with Annexin-V to assess apoptosis in the non-immune cell population by flow cytometry. The percentage of Annexin-V^+^ cells increased over time and was significantly higher in the discs harvested from NSG mice reconstituted with allogeneic human immune cells than controls (Figures 5E and 5F). Taken together, our results reveal that bioengineered kidney tissues express HLA molecules upon *in vivo* implantation, enhancing their immunogenicity. These data also show that our humanized mouse model can trigger transplant rejection of these engineered kidney tissues.

### *In vivo* kidney tissue rejection is inhibited by an immunosuppressive drug

**Figure 6:**
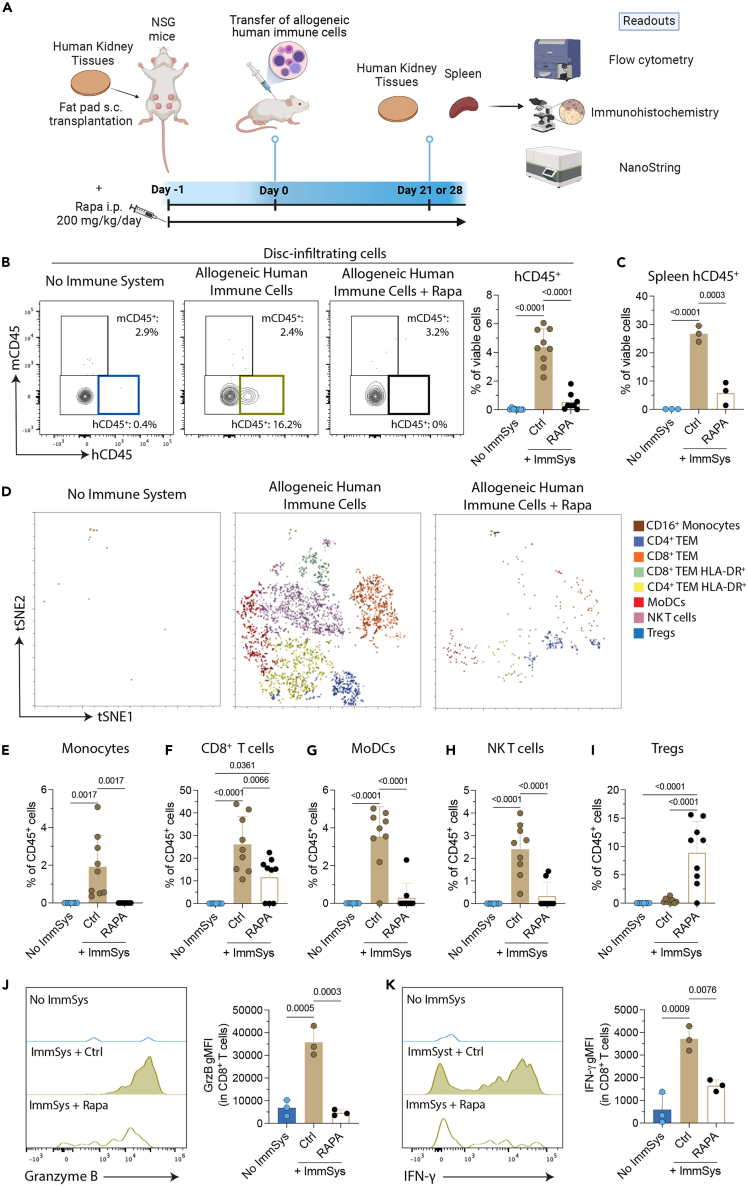
Rapamycin controls kidney tissue-effector cell immune response *in vivo* (A) Kidney tissues were subcutaneously (s.c.) transplanted into the fat pad of NSG mice reconstituted with or without allogeneic human immune cells. One cohort of NSG mice was intraperitoneally (i.p.) treated with 200 mg/kg/day of rapamycin (Rapa). The kidney tissues were recovered and then analyzed by histology, flow cytometry, and NanoString analysis on days 21 and 28. (B and C) Representative contour plots (gated on viable singlets) and percentages of the (B) kidney tissue-infiltrating cells and (C) splenic human CD45^+^ leukocytes. Data are representative of two independent experiments. Statistical analyses are done by one-way ANOVA with Tukey post-test (*n* = 7–9 tissues/group, and 3 spleens/group). (D–K) (D) viSNE plots represent the eight immune cell meta-clusters identified by FlowSOM in the three groups. Percentages of kidney tissue-infiltrating (E) human monocytes, (F) total CD8^+^ T cells, (G) monocyte-derived dendritic cells (MoDCs), (H) CD4^−^CD8^−^CD16^+^ T cells, and (I) regulatory T cells (Tregs) at day 21 after the transfer of allogeneic human immune cells. Data are representative of two independent experiments. Statistics are performed by one-way ANOVA with Tukey’s multiple comparisons test (*n* = 7–9 tissues/group). Expression of (J) granzyme B (GrzB) and (K) IFN-γ production by kidney tissue-infiltrating CD8^+^ T cells at day 21 after the transfer of allogeneic human immune cells. gMFI, geometric mean fluorescence intensity. Statistics are performed by one-way ANOVA with Tukey’s multiple comparisons test (*n* = 3 tissues/group).

The control of effector rejection responses with immunosuppressive drugs is crucial for the successful clinical translation of engineered tissues and, ultimately, whole organs. Indeed, developing models that mimic human kidney transplant rejection provides useful platforms for assessing novel therapeutic targets. Hence, we used our model to evaluate the effects of the immunosuppressive agent rapamycin (Rapa, sirolimus) in kidney tissue injury induced by allogeneic human immune cells. Inhibitors of the mammalian target of rapamycin (mTOR) pathway, like Rapa, are widely used as a class of maintenance immunosuppressive drugs after organ transplantation,^20^^,^^21^ and they have the benefit of not only inhibiting effector T cells but also of promoting regulatory T cells.^22^ We transplanted kidney tissues in NSG mice reconstituted with allogeneic human immune cells and administered Rapa daily (Figure 6A). Transplanted discs were analyzed by flow cytometry, histology, and NanoString on day 21, at the peak of their immune response. Host integration and vascularization were observed in all cases (Figure S5A), with their weights decreased in tissues harvested from NSG mice treated with Rapa (Figure S5B). Human and CD45^+^ leukocytes were also significantly decreased in the kidney tissues (Figure 6B) and spleens (Figure 6C) of NSG mice reconstituted with allogeneic human immune cells and treated with Rapa. These mice had smaller spleens with reduced weight (Figures S5C and S5D), suggesting an inhibition of the alloimmune response. The immune characterization of the human leukocytes infiltrating the transplanted tissues demonstrated that Rapa treatment significantly decreased tissue-infiltrating monocytes (Figures 6D and 6E), total and activated CD8^+^ T cells (Figures 6D and 6F), MoDCs (Figures 6D and 6G), and CD4^−^CD8^−^CD16^+^ T cells (Figures 6D and 6H). Interestingly, this treatment also increased the proportion of regulatory T cells (Tregs) in the tissues (Figures 6D and 6I). We also found that Rapa could control the production of the cytotoxic markers granzyme B (GrzB, Figure 6J) and IFN-γ (Figure 6K) in kidney tissue-infiltrating CD8^+^ T cells from NSG mice reconstituted with allogeneic human immune cells. Overall, our findings demonstrate that Rapa treatment can control innate and adaptive effector immune responses and proportionally increases Tregs *in vivo*.

**Figure 7:**
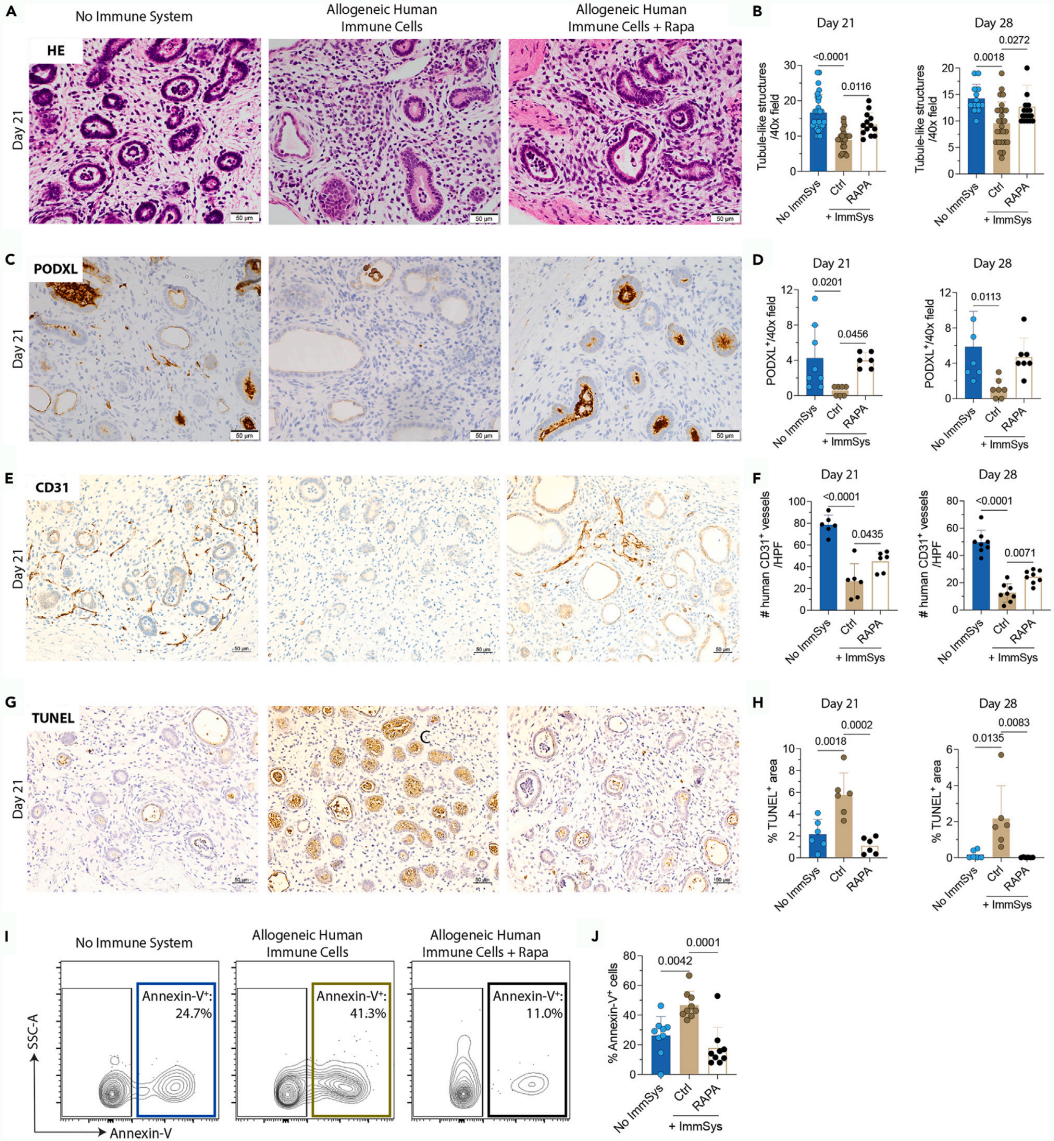
Rapamycin controls immune rejection of transplanted kidney organoid-based tissues NSG mice are kidney tissue discs-implanted as displayed in Figure 6A. Tissues are harvested and analyzed on days 21 and 28 after the transfer of allogeneic human immune cells. (A–H) (A and B) Representative H&E staining and quantification of tubuli-like structures in the three experimental groups. Immunostaining and quantification of (C and D) PODXL, (E and F) CD31, and (G and H) TUNEL assay in the three experimental groups. Scale bars, 50 μm. Statistic by one-way ANOVA with Tukey post-test (*n* = 6–28 tissues/group/time point). (I and J) (I) Representative contour plots and (J) quantification of Annexin-V^+^ (apoptotic) cells by flow cytometry on kidney discs harvested on day 21 after transferring allogeneic human immune cells. Statistic by one-way ANOVA with Tukey’s multiple comparisons test (*n* = 9 tissues/group). Data are representative of 2–3 independent experiments.

Next, we investigated whether Rapa treatment could maintain kidney tissue integrity on days 21 and 28. Tubule-like (Figures 7A and 7B; Figure S6A) and glomerular-like (PODXL^+^) structures (Figures 7C, 7D, and S6B) are significantly preserved over time when NSG mice with an allogeneic human immune system are treated with Rapa. This same pattern is observed for CDH1^+^ (Figures S6E–S6H) and WT-1^+^ structures (Figures S6I–S6L). We then evaluated whether Rapa interferes with host vascularization and inhibits tissue cell death induced by the allogenic human immune cells. Rapa significantly improves the numbers of CD31^+^ vessels in the kidney tissues on days 21 and 28 (Figures 7E, 7F, and S6C), reducing vascular endothelium injury. Apoptosis of kidney cells in these tissues is also significantly decreased in Rapa-treated mice, as confirmed by TUNEL (Figures 7G, 7H, and S6D) and Annexin-V (Figures 7I and 7J) stains. The observed changes in the phenotypes of infiltrating immune cells and suppression of kidney injury mimic the immunomodulatory effects of Rapa in the clinical setting. Our data suggest that the use of immunosuppressive drugs, like rapamycin, can prevent the rejection of the transplanted kidney tissues assembled from OBBs.

### Characterizing the kidney tissue microenvironment during rejection

**Figure 8:**
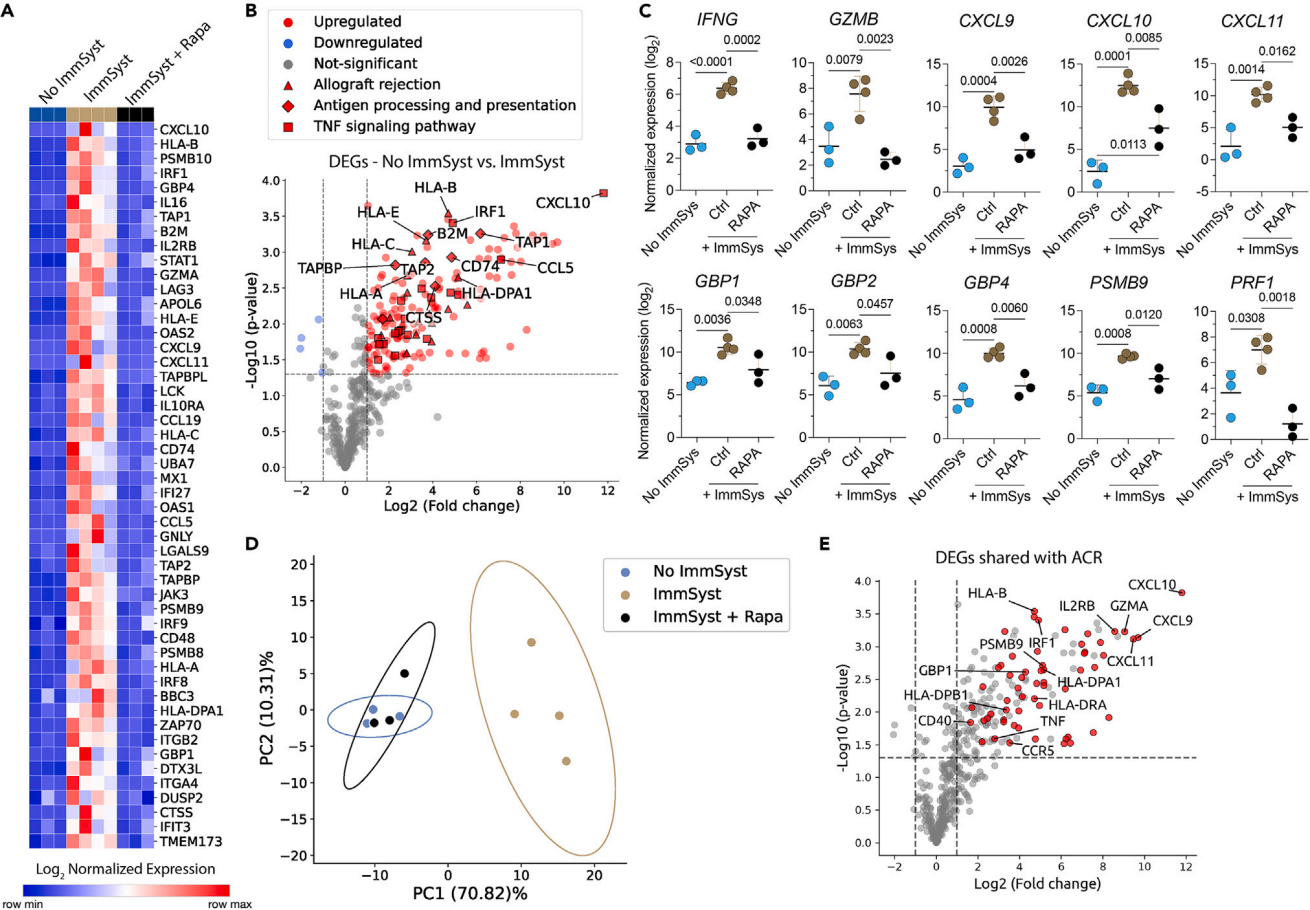
Rejection-related gene expression signature of transplanted kidney organoid-based tissues harvested from NSG mice reconstituted with allogeneic human immune cells Kidney tissues were transplanted into NSG mice reconstituted with (ImmSyst) or without (No ImmSyst) allogeneic human immune cells. One cohort of NSG mice was treated with 200 mg/kg/day of rapamycin (ImmSyst + Rapa). The kidney tissues were recovered and then analyzed by NanoString on day 21. (A) Heatmap of the top 50 differentially expressed genes (DEGs) in tissues from NSG mice reconstituted with allogeneic human immune cells (ImmSyst, *n* = 4) and those treated with Rapa (ImmSyst + Rapa, *n* = 3) compared with NSG mice with no immune system (No ImmSyst, *n* = 3). Data expressed as log2 normalized expression. (B) Volcano plot showing DEGs in the group ImmSyst in relation to the No ImmSyst group. Each dot represents an individual gene, and each shape represents a Kyoto Encyclopedia of Genes and Genomes (KEGG) pathway. Log2 fold change is represented on the x axis, and the y axis displays log10 of each gene’s *p* value. (C) Log2 normalized gene expression of IFN-γ-related transcripts in the No ImmSyst, ImmSyst, and ImmSyst + Rapa groups. Statistics used one-way ANOVA with Tukey’s multiple comparison test. (D) Unsupervised principal-component analysis of the top 166 DEGs, clustering the samples from NSG mice reconstituted with allogeneic human immune cells (ImmSyst) system compared with No ImmSyst and ImmSyst + Rapa events, which clustered together. (E) Volcano plots showing upregulated DEGs shared with human kidney transplant acute cellular rejection. Log_2_ fold change is represented on the x axis, and the y axis displays log_10_ of each gene’s *p* value.

To determine the molecular characteristics associated with kidney rejection, we compared the gene expression profiles of kidney tissues harvested from NSG mice with no immune system or reconstituted with allogeneic human immune cells treated or not with Rapa. Among 770 genes analyzed using the NanoString technology, 166 genes were differentially expressed during rejection (log2 fold change >1.5; adjusted *p* value <0.05). The top 50 differentially expressed genes (DEGs) are shown in Figure 8A. The complete list of DEGs is displayed in Tables S3 and S4. Of the 166 DEGs, 148 were upregulated in the allogeneic human immune cells group and included molecules related to allograft rejection, antigen processing and presentation, and tumor necrosis factor signaling pathway (Figure 8B).

In kidney transplant recipients, cellular rejection is associated with a gene signature related to IFN-γ responses.^23^^,^^24^ We analyzed whether the presence of allogeneic human immune cells would induce the expression of IFN-γ-related genes in the kidney tissues and how Rapa could modulate their expression. We found an increase in the expression of kidney tissues *IFNG*, *GZMB*, *CXCL9*, *CXCL10*, *CXCL11*, *GBP1*, *GBP2*, *GBP4*, *PSMB9*, and *PRF1* after the transfer of allogeneic human immune cells (Figure 8C). The expression of these transcripts was significantly reduced by the treatment with Rapa (Figure 8C). A principal-component analysis conducted using all 166 DEGs revealed distinct clustering. The gene signature of the kidney tissues harvested from mice with allogeneic human immune cells differed from those without an immune system. Notably, transplanted kidney tissues from NSG mice without an immune system and those reconstituted with allogeneic human immune cells and treated with Rapa clustered together (Figure 8D), confirming its ability to reduce effector immune responses. Finally, we compared the gene expression profile of the rejecting kidney tissues with molecular signatures of acute cellular rejection in human kidney transplants. As previously reported by our group,^25^ mRNA transcripts measured in kidney biopsy specimens during acute rejection were used. We found that the transcriptional profile of our kidney organoid-based tissues resembled the gene signature observed in human kidney transplant rejection (Figure 8E), especially an IFN-γ-related signature.

## Discussion

The engineering and transplantation of functional human kidney tissue could provide a potential alternative for treating patients with ESRD. We developed a fabrication method of kidney tissue discs comprising multiple kidney organoids, which were then transplanted subcutaneously into the fat pad of immunocompromised NSG mice. These tissues express PODXL (podocytes), CDH1 (distal tubule), LTL (proximal tubule), and CD31 (endothelial cells) *in vitro* and are able to engraft and survive *in vivo*. The presence of allogeneic human immune cells had harmful effects on the transplanted kidney tissues, including reducing their inherent vascularization (decreased expression of the CD31 endothelial marker) and enhancing cell death. A decrease in nephron epithelial markers, e.g., PODXL and CDH1, was also observed when transplanted kidney tissues were exposed to allogeneic human immune cells. Importantly, the rejection process exhibited similar molecular characteristics to those observed during kidney transplant rejection in humans. Importantly, rejection was suppressed upon administering the immunosuppressant, rapamycin. Our model serves as a platform for studying the immunogenicity of engineered transplanted tissues and new immunosuppressant drugs under development for kidney transplant recipients.

Kidney replacement therapy consists of life-supporting interventions to restore kidney function in patients with ESRD. While kidney transplantation is the most effective kidney replacement therapy, an insufficient supply of transplantable donor kidneys requires the majority of patients with renal failure to undergo dialysis, which mechanically filters waste products and excess fluids from the blood. However, dialysis is associated with severe morbidity and mortality, in particular, cardiovascular disease.^4^^,^^5^ Numerous strategies are being explored for the development of transplantable kidneys, including xenotransplantation, decellularization and recellularization of scaffolds, kidney organoid-based therapies, and interspecies blastocyst complementation.^17^ Kidney organoids derived from patient-specific hiPSCs are already serving as valuable tools for studying kidney development and diseases. Our study provides a foundational step toward using kidney organoids as building blocks for biomanufacturing transplantable kidney tissues, which may restore partial or complete function.

By understanding the role of HLA mismatches that serve as the main antigens triggering alloimmune responses during transplantation, we can create engineered kidney tissues that are more resistant to rejection. While HLA molecules have lower expression in kidney organoids *in vitro*, we observed an increased expression of both class I and II upon transplantation and in the presence of allogeneic human immune cells. Moreover, transcriptional analyses of the kidney tissues in NSG mice reconstituted with allogeneic human immune cells revealed a similar molecular signature as observed in biopsies of kidney transplant recipients with acute cellular rejection with dominant IFN-γ-related transcripts.^23^^,^^24^ Furthermore, evidence of target kidney tissue damage was evident, confirming the immune-mediated injury. We found that rapamycin inhibits the effector alloimmune response and mitigates kidney tissue injury.

In summary, our ability to scalably create kidney organoid-based tissues has advanced our understanding of the immunological challenge of transplanting HLA-incompatible tissues. Our observations underscore the importance of applying therapeutic approaches to minimize this alloimmune response via the use of immunosuppressive drugs or autologous or hypoimmunogenic stem cells for organoid and tissue generation.

### Limitations of the study

Limitations of our study include that our model only mimics acute cellular rejection, which is the dominant mode of rejection during the first year post-transplantation, since PBMCs do not fully recapitulate all components of the human immune system, such as the engraftment of B cells and antibody production. Moreover, we plan to implant engineered kidney tissues with embedded vascular and drainage networks and assess their *in vivo* function looking ahead.

## Resource availability

### Lead contact

Further information and requests for resources and reagents should be directed to and will be fulfilled by the lead contact, Dr. Leonardo V. Riella (lriella@mgh.harvard.edu).

### Materials availability

This study did not generate new unique reagents.

### Data and code availability

• NanoString data have been deposited at GEO and are publicly available as of the date of publication. Accession numbers are listed in the key resources table.
• This paper does not report the original code.
• All other data reported in this paper will be shared by the lead contact upon request.
• Any additional information required to reanalyze the data reported in this paper is available from the lead contact upon request.

## Acknowledgments

This work was supported by the Wellcome LEAP Human Organs, Physiology, and Engineering (HOPE) program, the NIH Re(Building) a Kidney Consortium (NIH UC2DK126023 to J.A.L.), the NIH award DP2EB029388/DK133821 to R.M., and NIH grants R56DK122380 to J.A.L. and R01AI143887-05 to L.V.R. We thank P. Stankey for assistance in printing the 3D stamps; A. Moisan, K. Wolf, K. Kroll, D. Reynolds, N. Gupta, and M. Creighton for insightful discussions; the Harvard Center for Biological Imaging for experimental assistance; and Kazumi Ida and Chengcheng Zhang for technical assistance in kidney organoid differentiation.

## Author contributions

T.J.B., J.O.A., R.v.G., S.G.M.U., R.M., J.A.L., and L.V.R. designed the research. T.J.B., Y.G., and R.B.G. performed disc implantation, harvesting, tissue processing, and flow cytometry experiments. T.J.B. and K.L. analyzed the flow cytometry experiments. T.J.B. and G.T.R. performed transcriptomics experiments and analyses. T.J.B., J.O.A., K.K., M.T., K.H., and I.A.R. performed histological analyses. J.O.A., K.K., M.T., K.H., and R.M. developed the direct 3D differentiation approaches using stirred bioreactors. J.O.A., K.K., M.T., K.H., and J.E.R. generated kidney organoids for the discs. J.O.A. and S.G.M.U. conceived, designed, and implemented the tissue disc stamping approach used to create uniform discs. J.O.A. and R.v.G. casted, cultured, and characterized the tissue discs prior to implantation. T.J.B., Y.G., J.O.A., J.A.L., and L.V.R. wrote the manuscript. All authors edited the manuscript.

## Declaration of interests

J.A.L. serves on the Scientific Advisory Board of Trestle Biotherapeutics. R.M. is an inventor on a patent related to this work filed by the President and Fellows of Harvard College and Mass General Brigham (PCT/US2018/036677 licensed to Trestle Biotherapeutics). R.M. holds a stock option in Trestle Biotherapeutics. R.M. served as a consultant to Toray Industries and Ajinomoto.

## STAR★Methods

### Key resources table

**Table:** 

REAGENT or RESOURCE	SOURCE	IDENTIFIER
**Antibodies**		

Anti-human CD8-BUV737	BD Biosciences	clone SK1; RRID: AB_2870086
Anti-human CD4-BUV395	BD Biosciences	clone SK3; RRID: AB_2738273
Anti-human CD19-BV785	Biolegend	clone HIB19; RRID: AB_2563442
Anti-human CD123-BV711	Biolegend	clone 6H6; RRID: AB_2566354
Anti-human CD27-BV650	Biolegend	clone O323; RRID: AB_2562096
Anti-human CD3-BV605	Biolegend	clone OKT3; RRID: AB_2561911
Anti-human CD14-BV510	Biolegend	clone M5E2; RRID: AB_2561946
Anti-human CD66b-BV421	Biolegend	clone 6/40c
Anti-human CD127-PerCP-Cy5.5	Biolegend	clone A019D5; RRID: AB_10897104
Anti-human CD16-FITC	Biolegend	clone 368; RRID: AB_314206
Anti-human CD56-PE-Cy7	Biolegend	clone 5.1H11; RRID: AB_2563927
Anti-human CD56-AF700	Biolegend	clone 5.1H11; RRID: AB_2564099
Anti-human CD45-PE-Cy5	Biolegend	clone HI30; RRID: AB_314398
Anti-human CCR7-PE-CF594	Biolegend	clone G043H7; RRID: AB_2563641
Anti-human CD25-PE	Biolegend	clone M-A251; RRID: AB_2561861
Anti-human HLA-DR-APC-Cy7	Biolegend	clone L243; RRID: AB_493586
Anti-human HLA-DR-BV650	Biolegend	clone L243; RRID: AB_2563828
Anti-human CD11c-AF700	Biolegend	clone 3.9; RRID: AB_2819923
Anti-human CD45RA-APC	Biolegend	clone HI100; RRID: AB_314416
Anti-human HLA-A/B/C-BV605	Biolegend	clone W6/32; RRID: AB_2566151
Anti-human HLA-A/B/C-APC	Biolegend	clone W6/32; RRID: AB_314879
Anti-human Ki67-BV605	Biolegend	clone Ki-67; RRID: AB_2563863
Anti-human Ki67-BV711	Biolegend	clone Ki-67; RRID: AB_2563861
Anti-human granzyme B-PE	Biolegend	clone QA18A28; RRID: AB_2801075
Anti-human IFN-γ-BV421	Biolegend	clone 4S.B3; RRID: AB_2561398
Anti-human TRA-1-60-FITC	BD Biosciences	clone TRA-1-60; RRID: AB_1645492
Anti-human SSEA-4-AF647	BD Biosciences	clone MC813-70; RRID: AB_2033991
Anti-human Oct3/4-BV421	BD Biosciences	clone 40/Oct-3; RRID: AB_2739320
Anti-mouse CD45-BV421	Biolegend	clone 30-F11; RRID: AB_2562559
TruStain FcX, anti-mouse CD16/32	Biolegend	clone 93; RRID: AB_1574975
TruStain FcX, Fc receptor blocking solution	Biolegend	Cat # 422302; RRID: AB_2818986
Anti-human PODXL	Abcam	clone EPR9518; RRID: AB_2895107
Anti-human CDH1	Cell Signaling	clone 24E10; RRID: AB_2291471
Anti-human CD4	Biocare	clone 4B12
Anti-human CD8	Biocare	clone C8/144B; RRID: AB_1792537
Anti-human K18	Abcam	clone EPR1626; RRID: AB_11155892
Anti-human HLA class II-DR/DP/DQ	Abcam	clone CR3/43; RRID: AB_306142
Anti-human HLA class I A/B/C	Abcam	clone EMR8-5; RRID: AB_1269092
Anti-human PODXL	R&D Systems	clone AF1658; RRID: AB_354920
Anti-human CDH1	Abcam	Cat # ab40772; RRID: AB_731493
Anti-human CD31	Abcam	Cat # ab9498; RRID: AB_307284
Anti-human CD31	Abcam	Cat # ab28364; RRID: AB_726362

**Chemicals, peptides, and recombinant proteins**		

Annexin V-BV510	Biolegend	Cat # 640937
Annexin V binding buffer	Biolegend	Cat # 422201
LIVE/DEAD™ Fixable Blue Dead Cell Stain Kit	Thermo Fisher	Cat #L34961
StemFit02 medium	Ajinomoto Co., Inc.	Cat # ASB01
mTeSR™ Plus medium	STEMCELL Technologies	Cat # 100–0276, 100-1130
Matrigel	Corning	Cat # 47743-720
DMEM/F-12 medium	Thermo Fisher	Cat # 11320033
DPBS, no calcium, no magnesium	Thermo Fisher	Cat # 14190144
ACCUTASE™	STEMCELL Technologies	Cat # 07922
ReLeSR™	STEMCELL Technologies	Cat # 100-0484
Gelatin from porcine skin (Type A, 300 bloom form)	Sigma-Aldrich	Cat #G1890
Thrombin	Sigma-Aldrich	Cat #T4648-10KU
Pluronic F127	BASF	Cat # 6361
Fibrinogen, Bovine Plasma	Millipore	Cat # 341573
Advanced RPMI 1640 Medium	Thermo Fisher	Cat # 12633012
GlutaMAX™ Supplement	Thermo Fisher	Cat # 35050061
Antibiotic-Antimycotic	Thermo Fisher	Cat # 15240062
FBS	Thermo Fisher	Cat # 16000044
Aminocaproic acid	Thermo Fisher	Cat # 103305000
0.025% trypsin with EDTA	Thermo Fisher	Cat # 25200056
Collagenase Type IV	STEMCELL Technologies	Cat # 07909
Recombinant human IFN-γ	R&D Systems	Cat # 285-IF
Rapamycin	Millipore	Cat # 553211
Lotus Tetragonolobus Lectin (LTL), Biotinylated	Vector	Cat # B-1325-2

**Critical commercial assays**		

RNeasy FFPE Kit	Qiagen	Cat # 73504
nCounter Standard Master Kit	NanoString	Cat # NAA-AKIT-01
ApopTag® Peroxidase *In Situ* Apoptosis Detection Kit	Millipore	Cat # S7100
ELISPOT Human IFN-γ ELISPOT Pair	BD Biosciences	Cat # 551873

**Deposited data**		

Raw NanoString data	This paper	GEO: GSE242885

**Experimental models: Cell lines**		

Human: BJFF human-induced PSCs	Laboratory of Prof. Sanjay Jain	N/A

**Experimental models: Organisms/strains**		

PBMCs from healthy volunteers	This study (MGH)	N/A
NSG (*M. musculus*)	Jackson Laboratory	NOD.Cg-Prkdc^scid^ Il2rg^tm1Wjl^/SzJ

**Oligonucleotides**		

nCounter PanCancer Immune Profiling Gene Expression (GX) Codeset	NanoString	Cat # XT-CSO-HIP1-12

**Software and algorithms**		

FlowJo v 10.7.1	BD Biosciences	N/A
Graphpad Prism v9.0	GraphPad Software	N/A
nSolver Analysis Software v4.0.70	Nanostring	N/A
Eplets mismatches calculator	Duquesnoy et al.^26^	https://www.epregistry.com.br/calculator/index.html
cellSens Imaging Software	Olympus	N/A
FlowSOM plugin (v2.6)	BD Biosciences	N/A
Cluster Explorer plugin (v1.6.3)	BD Biosciences	N/A
Python 3.9.16	Python	N/A
Pandas 1.3.5	McKinney et al.^27^	https://zenodo.org/search?q=conceptrecid%3A%223509134%22&l=list&p=1&s=10&sort=-version
Matplotlib 3.7.1	Hunter et al.^28^	https://zenodo.org/records/10951225
Morpheus	https://software.broadinstitute.org/morpheus	N/A

**Other**		

Keyence VHX-2000 zoom microscope	Keyence	N/A
Axio Imager.M2	ZEISS	N/A
Fortessa X-20	BD Biosciences	N/A
VENTANA BenchMark Stain System	Roche	N/A
nCOUNTER FLEX	NanoString	N/A
ImmunoSpot analyzer	Cellular Technology	N/A
Olympus BX53 microscope with a DP27 camera	Olympus	N/A
Leica Zeiss LSM 880 + FLIM microscope	Zeiss	N/A
2100 Bioanalyzer system	Agilent	N/A

### Experimental model and study participant details

#### hiPSC culture and kidney organoid differentiation

BJFF.6 human-induced PSCs (provided by Prof. Sanjay Jain at Washington University) are maintained in either StemFit02 (Ajinomoto Co., Inc.) or mTeSR plus (STEMCELL Technologies) media and used up to passage 50. For maintenance, BJFF iPSCs were passaged upon reaching ∼50–70% confluency. BJFFs are seeded into new plates/flasks coated with 1% Matrigel (Corning) in DMEM/F12 medium (Thermo Fisher), as previously described.^7^ BJFFs are lifted from the original plate/flask by removing the stem cell medium, washing 1X with DPBS without Ca^2+^ and Mg^2+^ (Gibco), incubating with Accutace or ReLeSR (both from STEMCELL Technologies) for 5–10 min at 37°C, and then using stem cell media to collect the lifted iPS cells. The BJFFs are then seeded into the new, pre-coated, plates/flasks in StemFit02 or mTeSR plus medium. Medium is changed no later than every 48-72h.

Before starting kidney organoid differentiation, BJFFs are grown to 80% confluency and lifted into a single cell suspension by removing the stem cell medium, washing 1X with PBS without Ca^2+^ and Mg^2+^, and incubating with Accutace or ReLeSR (STEMCELL Technologies) for 5–10 min at 37°C. The single-cell suspension is then seeded into stirred bioreactors (Reprocell) or spinner flasks at a density of 500k to 1 million cells per mL and spun at a rate of 65–80 rpm for 48h, leading to the spheroid formation. After forming spheroids, kidney organoids are differentiated using a directed differentiation method as previously described.^7^

#### Mice

NSG (NOD.Cg-Prkdc^scid^ Il2rg^tm1Wjl^/SzJ) mice were purchased from the Jackson Laboratory and maintained as breeding colonies in the Massachusetts General Hospital animal facility with water and food *ad libitum*. All mice used in the experiments were 8–12 weeks old. Following the Institutional Animal Care and Use Committee (IACUC) and National Institutes of Health (NIH) Animal Care guidelines, mice were bred and housed in individual and standard mini-isolators under specific pathogen-free conditions. The Mass General Brigham IACUC approved all experiments under protocol number 2020N000125.

#### Peripheral blood mononuclear cells (PBMCs) and human T cells isolation

PBMCs were used as allogeneic human immune cells. PBMCs were isolated from peripheral blood samples of a healthy volunteer by density gradient centrifugation using Ficoll-Paque solution (GE Healthcare) and were freshly transferred to NSG mice. Human total T cells (CD3^+^) were magniticaly isolated by negative selection using MojoSort Human CD3 T cell Isolation Kit from Biolegend. These experiments were approved under the Mass General Brigham IRB number 2017P000298.

### Method details

#### Kidney tissue assembly

Each kidney tissue disc is assembled by depositing a densely cellular matrix composed of kidney organoid building blocks in a fibrinogen solution into cylindrical cavities stamped in an extracellular matrix composed of 8 wt % gelatin, 2.5 mM CaCl_2_, and 10 U thrombin, in DMEM/F12 medium. The gelatin is made by first preparing a 15% (w/v) gelatin solution (Type A, 300 bloom form porcine skin, Sigma-Aldrich) by adding prewarmed PBS without Ca^2+^ and Mg^2+^ to the gelatin powder. This gelatin solution is stirred for 12 h at 70°C to allow for the complete dissolving of the gelatin. A 250 mM CaCl_2_ stock solution is made by dissolving CaCl_2_ pellets in sterile water and storing them at 4°C for long-term storage. 2,000 U/mL stock solutions of thrombin are created by reconstituting lyophilized thrombin (Sigma-Aldrich) in sterile water and storing it at − 20°C. After warming all components to 37°C, the constituents are mixed together in the following order: DMEM/F12, gelatin, CaCl_2_, and thrombin. The gelatin-thrombin solution is held at 37°C for 5-10 min to ensure proper mixing prior to casting into a PDMS reservoir. Next, a disc stamp produced by stereolithography, a 3D printing method, using htm140v2 resin or Biomed clear resin and treated with 5 wt % Pluronic F127 (BASF) to prevent adhesion, is placed on top of the PDMS reservoir containing the gelatin-thrombin mixture. The stamp and PDMS reservoir containing the gelatin-thrombin solution are allowed to cool at 4°C for at least 30 min to solidify this gel. The 3D-printed stamp is then carefully removed, leaving behind the cylindrical cavities that serve as individual molds for each tissue disc. Each mold is transferred to an ultra-low adhesion 6-well plate using an 8 mm biopsy punch and spatula when they are held at 4°C for no longer than 24h prior to tissue disc casting.

Before casting, kidney organoids are collected from the stirred bioreactors using a 10mL serological pipette, passed through a 500 μm cell strainer to remove larger aggregates, and allowed to settle at the bottom of a conical tube. A fibrinogen mixture is created containing: 10 mg/mL fibrinogen and 2.5 mM CaCl_2_ in DMEM/F12 media. The fibrinogen stock solution is made by first preparing a 50 mg/mL stock from lyophilized bovine blood plasma protein (Millipore). It is reconstituted in a controlled manner by adding sterile PBS without Ca^2+^ and Mg^2+^ and keeping it at 37°C without agitation for 2–3h. Once complete, the fibrinogen solution is stored in smaller aliquots at − 20°C for later use. The CaCl_2_ stock solution is described using the previously described process. Once the fibrinogen and CaCl_2_ are properly mixed into the DMEM/F12 to create the fibrinogen mixture, excess media is removed. The organoids are then mixed with 3x the volume of the fibrinogen mixture. This organoid-laden fibrinogen solution is allowed to settle for ∼2 min and the supernatant is removed. The remaining organoids are then mixed with 1x volume of fibrinogen mixture and centrifuged at 30 rcf for 3 min 200 μL Eppendorf Combitip advanced positive displacement tips (Eppendorf) are cut with a razor blade to make a wide bore opening on the depositing end and loaded with small volumes of 5 wt % gelatin in PBS to protect the organoids from the displacement plunger. The tips are loaded with the organoid-fibrinogen and then centrifuged in a custom 3D printed holder at 30 rcf for 3 min to create a compacted organoid-fibrinogen tissue matrix. Once compacted, an Eppendorf Repeater M4 multi-dispenser pipette is used to deposit 8 μL of this compacted tissue matrix into each disc mold, where it resides for ∼5 min to allow thrombin diffusion from the surrounding gelatin matrix that drives polymerization of the fibrin gel, i.e., which “binds” the organoids together. A droplet of the gelatin-thrombin mixture is then placed on top of each disc to ensure the polymerization of that region. 5 mL of Advanced RPMI media with 1% GlutaMAX, 1.5% FBS, 1% anti-anti, and 1% aminocaproic acid (ACA) is then added to each well and placed on an orbital shaker at 90 rpm in the incubator. Full-volume media changes are performed 24h after disc formation and each subsequent 48h over a 7-day culture period prior to implantation. The kidney tissue discs are live-imaged at days 0, 4, and 7 using a Keyence zoom microscope (VHX-2000, Keyence, Japan).

#### HLA typing and eplet mismatches calculations

Allogeneic human immune cells (PBMCs) and iPSCs (BJFF cells) were HLA typed by the Histogenetics company using a 4x resolution (class I: exons 1–8 typed, and class II exons 2–6 typed). Eplets mismatches were calculated using the calculator from the HLA Eplet Registry (https://www.epregistry.com.br/calculator/index.html).

#### Co-cultures of T and kidney organoid cells

We obtained single cell suspensions from kidney organoids in a two-step protocol based on.^29^ Briefly, differentiated kidney organoids were collected on 15 mL tubes, span down at 400 g and the pellet was incubated with 10 mL of 0.025% trypsin with EDTA (Thermo Fisher) for 3 min at 37°C. Samples were washed, span down at 400 g and the supernatant removed. After that, the cell pellet was incubated with 2 mL of 1 mg/mL collagenase IV solution (STEMCELL Technologies) for 20 min at 37°C. The pellet was disaggregated into single cells by gentle pipetting using a P1000 pipette. Once the cell solution no longer contained visible aggregates, 3.5 mL of PBS containing calcium and magnesium was added. Cells were then filtered through a 35 μm mesh. After a span down at 400 g, cells were resuspended in AdvRPMI (Thermo Fisher) with 1X GlutaMax (Thermo Fisher), 1.5% fetal bovine serum (Gibco), and 1% Antibiotic/Antimycotic (Gibco) medium (culture medium).

To stimulate the expression of HLA molecules, kidney cells were stimulated with 500 U/ml of recombinant human IFN-γ (R&D Systems) for 2 days. In a set of experiments, cells were stained for HLA-A/B/C and HLA-DR and analyzed by flow cytometry. For the co-culture experiments, kidney cells were washed twice, resuspended in culture medium, and irradiated (30 Gy). IFN-γ-stimulated and irradiated kidney cells were then co-cultured at different ratios with 2 × 10^5^ purified human allogeneic T cells (0:1, 1:10, 1:5, 1:1 – organoid:T cell ratio) for 3 days at 37°C, 5% CO_2_. Cell death within the CD45^−^ population and T cell activation markers were evaluated by flow cytometry.

#### ELISPOT assay

Human IFN-γ ELISPOT assay was performed using a kit from BD Biosciences. Briefly, 0.45 μm hydrophobic, high-protein-binding Immobilon-P membrane plates (Millipore) were coated with anti-human IFN-γ capture antibody at 4°C overnight, followed by blocking with culture medium for 1 h at room temperature. The plates were washed twice with PBS 1x, and IFN-γ-stimulated and irradiated kidney cells were then co-cultured at different ratios with 2 × 10^5^ purified human allogeneic T cells (0:1, 1:10, 1:5, 1:1 – organoid:T cell ratio) for 3 days at 37°C, 5% CO_2_. The assay was developed according to the manufacturer’s protocol, and spots were detected and counted using an ImmunoSpot analyzer (Cellular Technology).

#### Kidney tissue implantation

NSG recipient mice were subcutaneously transplanted with ∼10 mm^3^ human kidney organoid discs. Discs were surgically positioned into the fat pad on the abdomen. On the next day, 13 × 10^6^ freshly isolated human PBMCs (allogeneic human immune cells) were transferred to tissue-implanted NSG mice via retro-orbital injection of the venous sinus. One cohort of NSG mice was transplanted with the kidney organoid discs but did not receive allogeneic human immune cells (PBMCs) as a negative control. Transplanted kidney tissues and spleens were procured on days 4, 14, 21 and 28 after the transfer of allogeneic human immune cells (Figure S1) for flow cytometry, histology and transcriptional analyses. To isolate cells for single-cell suspension, the discs and spleens tissues were mechanically dissociated through a 70 μm cell strainer, and RBCs were lysed using hypotonic ACK buffer (Lonza). In some sets of experiments, NSG mice were treated with 200 mg/kg/day (i.p.) with the immunosuppressive agent Rapamycin (Rapa, Millipore) for 21 or 28 days.

The percentage of human CD45^+^ cell engraftment varies according to the time point time analyzed. We have a mean of 2.3 ± 1.6% for day 4, 17.2 ± 6% for day 14, 40.7 ± 16.3% for day 21 and 74.5 ± 8.9% for day 28. Criteria for inclusion are ≥5% for day 14, ≥10% for day 21 and ≥20% for day 28.

#### Flow cytometry

We stained the isolated kidney cells and splenocytes for flow cytometry. Isolated cells were mouse (TruStain FcX, anti-mouse CD16/32, clone 93, Biolegend) and human (TruStain FcX, Fc receptor blocking solution, Biolegend) Fc-blocked for 20 min before staining for surface markers for 30 min in FACS buffer (2% FBS in PBS 1x) on ice. We used the following anti-human antibodies: CD8-BUV737 (1:50, clone SK1, BD Biosciences), CD4-BUV395 (1:50, clone SK3, BD Biosciences), CD19-BV786 (1:50, clone HIB19, Biolegend), CD123-BV711 (1:20, clone 6H6, Biolegend), CD27-BV650 (1:50, clone O323, Biolegend), CD3-BV605 (1:50, clone OKT3, Biolegend), CD14-BV510 (1:25, clone M5E2, Biolegend), CD66b-BV421 (1:50, clone 6/40c, Biolegend), CD127-PerCP-Cy5.5 (1:20, clone A019D5, Biolegend), CD16-FITC (1:40, clone 368, Biolegend), CD56-PE-Cy7 (1:50, clone 5.1H11, Biolegend), CD56-AF700 (1:50, clone 5.1H11, Biolegend), CD45-PE-Cy5 (1:100, clone HI30, Biolegend), CCR7-PE-CF594 (1:20, clone G043H7, Biolegend), CD25-PE (1:20, clone M-A251, Biolegend), HLA-DR-APC-Cy7 (1:50, clone L243, Biolegend), HLA-DR-BV650 (1:50, clone L243, Biolegend), CD11c-AF700 (1:25, clone 3.9, Biolegend), CD45RA-APC (1:50, clone HI100, Biolegend), HLA-A/B/C-BV605 (1:20, clone W6/32, Biolegend), HLA-A/B/C-APC (1:20, clone W6/32, Biolegend), Ki67-BV605 (1:33, clone Ki-67, Biolegend), Ki67-BV711 (1:33, clone Ki-67, Biolegend), granzyme B-PE (1:20, clone QA18A28, Biolegend), IFN-γ-BV421 (1:20, clone 4S.B3, Biolegend), TRA-1-60-FITC (1:20, clone TRA-1-60, BD Biosciences), SSEA-4-APC (1:5, clone MC813-70, BD Biosciences), Oct3/4-BV421 (1:50, clone 40/Oct-3, BD Biosciences). We used the following anti-mouse antibodies: CD45-BV421 (1:100, clone 30-F11, Biolegend). After surface staining, cells were stained for Annexin V-BV510 (1:50, Biolegend) in Annexin V binding buffer (Biolegend) for 15 min at room temperature. For intracellular staining, cells were fixed and permeabilized with the eBioscience Foxp3/Transcription factor Staining buffer set according to the manufacturer’s instruction (ThermoFisher) and stained with indicated antibodies overnight at 4°C. Stained cells were analyzed on an LSR Fortessa X-20 flow cytometer (BD Biosciences) with FACSDiva software (BD Biosciences). Data were analyzed with FlowJo software (TreeStar). Viable cells were selected based on the staining with LIVE/DEAD Fixable Blue Dead Cell Stain Kit (1:1000, Thermo Fisher) prior to the Fc-blocking.

#### Immunohistochemistry and immunofluorescence imaging

All specimens were formalin-fixed, processed and paraffin-embedded. Five μm-thick sections were prepared and stained with hematoxylin and eosin or were used for immunohistochemical studies. For some stains (i.e., CD31 and TUNEL assay), immunohistochemical staining was performed using an automated Ventana BenchMark Stain System (Roche). Antibodies used were rabbit polyclonal anti-human CD31 (Abcam). The TUNEL assay was performed using the ApopTag Peroxidase *In Situ* Apoptosis Detection Kit from Millipore. Stained sections were photographed using an Axio Imager M2 microscope (Zeiss) and processed with ImageJ and Photoshop software.

Other antibodies used were rabbit monoclonal anti-human podocalyxin (anti-PODXL, Abcam, clone EPR9518), rabbit monoclonal anti-e-cadherin (anti-CDH1, Cell Signaling, clone 24E10), rabbit monoclonal anti-Cytokeratin 18 antibody (anti-K18, Abcam, clone EPR1626), mouse monoclonal anti-human CD4 (Biocare, clone 4B12), mouse monoclonal anti-human CD8 (Biocare, clone C8/144B), mouse monoclonal anti-HLA class II-DR/DP/DQ (Abcam, clone CR3/43), mouse monoclonal anti-HLA class I A/B/C (Abcam, clone EMR8-5). Photomicrographs were taken using an Olympus BX53 microscope with a DP27 camera and cellSens Imaging Software (Olympus Life Science Solutions).

We used a Leica Zeiss LSM 880 + FLIM microscope to carry out all immunofluorescence confocal imaging. Harvested tissue discs were formalin-fixed and placed overnight at 4°C in a blocking solution containing 0.125% Triton X-100 and 1 wt % donkey serum in DPBS with calcium and magnesium. The blocking solution was washed off using DPBS, and primary antibodies were added for 24–48 h at 4°C in a staining solution containing 0.5 wt % BSA and 0.125% Triton X-100 in DPBS. Primary antibodies were washed off using DPBS and secondary antibodies were added using the staining solution described above. The primary antibody list used for the stains is provided in Table S2. DAPI (405), Alexa Fluor 488, Alexa Fluor 555, or Alexa Fluor 647 were used for secondary antibodies. Fibrin confocal imaging was conducted by imaging autofluorescence in the 555 channel, which was coupled with the PODXL antibody. ImageJ software was used for confocal image processing.

#### RNA extraction

We obtained six consecutive 10 μm sections from formalin-fixed paraffin-embedded (FFPE) kidney tissues harvested at different time points. Deparaffinization with Xylene and RNA extraction was performed in sterile 1.5 mL microcentrifuge tubes with the RNeasy FFPE Kit (Qiagen), according to the manufacturer’s instructions. The concentration and purity of the isolated total RNA were measured using the 2100 Bioanalyzer system (Agilent) at the Center for Advanced Molecular Diagnostics (CAMD) Research Core of the Brigham and Women’s Hospital. The absorbance ratio at 260/280 was used to determine RNA quality.

#### NanoString nCounter assay for mRNA gene expression assay

We analyzed 770 genes with the NanoString nCounter PanCancer Immune Profiling Gene Expression (GX) Codeset. Gene expression was measured on 100–200 ng of extracted RNA. Samples were processed on the NanoString nCounter Analysis System (NanoString Technologies) following the manufacturer’s instructions at the CAMD Research Core of the Brigham and Women’s Hospital.

### Quantification and statistical analyses

For independent 2-group comparisons, we used the unpaired 2-tailed Student’s t test for analysis. For multiple group comparisons, the 1-way ANOVA or two-way ANOVA test was used to determine differences, depending on the number of comparison groups. Multiple comparisons between levels were checked with the Tukey post hoc test for 1-way ANOVA and Šídák’s test for a two-way ANOVA. Differences were considered significant at *p* ≤ 0.05. Prism software was used for data analysis and drawing graphs (GraphPad Software, Inc.).

For flow cytometry, t-SNE and FlowSOM analyses were performed on FlowJo (v.10.8.1). Cell populations were downsampled from viable singlets human CD45^+^ cells using the Downsample (v.3.3) plugin for Flowjo, followed by concatenation. We then performed FlowSOM clustering on the concatenated file with the FlowSOM plugin (v2.6) was performed on the concatenated file with *n* = 8–9 clusters for the human immune cell populations. The FlowSOM output was surface markers expression and the percentage of cells in each cluster was used to calculate absolute numbers. The t-SNE maps were built with a vantage point tree algorithm with 1000 iterations. Cluster identification in the map was performed with the help of the Cluster Explorer plugin (v1.6.3) through the heatmap expression of surface markers CD16, CD127, CD66b, CD14, CD3, CD27, CD123, CD19, CD4, CD8, CD45RA, CD11c, HLA-DR, CD25, CCR7, and CD56.

For Nanostring analyses, we normalized raw gene expression counts and performed background correction, quality control and analyses with the nSolver Analysis Software (Version 4.0.70). Ten reference genes (*POLR2A*, *SDHA*, *SF3A1*, *TMUB2*, *NRDE2*, *MRPL19*, *PUM1*, *DNAJC14*, *STK11IP* and *ERCC3*) were used for normalization. We used the quality control parameters recommended by the manufacturer. To compare our gene signature with transcripts in acute cellular rejection in human kidney graft biopsies, we selected the upregulated genes from Win et al.^25^ and van der Zwan et al.^30^ and intersected them with the upregulated DEGs from this study. We defined upregulated genes as those with log2 fold change greater than 1.5 and a *p*-value less than or equal to 0.05. Some analyses were performed with Python 3.9.16, pandas 1.3.5 and matplotlib 3.7.1. The data discussed in this manuscript have been deposited in NCBI’s GEO and are accessible through GEO Series accession number GSE242885 (https://www.ncbi.nlm.nih.gov/geo/query/acc.cgi?acc=GSE242885).

## Supplemental information

Document S1. Figures S1–S6 and Tables S1, S2, and S4

Table S3. List of differently expressed genes in transplanted kidney tissues from NSG mice reconstituted with versus without allogeneic human immune cells, related to Figure 8
