# Supplementary material for: Exploring immune response toward transplanted human kidney tissues assembled from organoid building blocks
Source: iScience. 2024 Sep 13;27(10):110957. doi: 10.1016/j.isci.2024.110957 (PMC11471229; doi:10.1016/j.isci.2024.110957)
Supplement: Document S1. Figures S1–S6 and Tables S1, S2, and S4 [file mmc1.pdf]

iScience, Volume *27*

Supplemental information

Exploring immune response toward transplanted human kidney tissues assembled from organoid building blocks

Thiago J. Borges, Yoshikazu Ganchiku, Jeffrey O. Aceves, Ronald van Gaal, Sebastien G.M. Uzel, Ivy A. Rosales, Jonathan E. Rubins, Kenichi Kobayashi, Ken Hiratsuka, Murat Tekguc, Guilherme T. Ribas, Karina Lima, Rodrigo B. Gassen, Ryuji Morizane, Jennifer A. Lewis, and Leonardo V. Riella

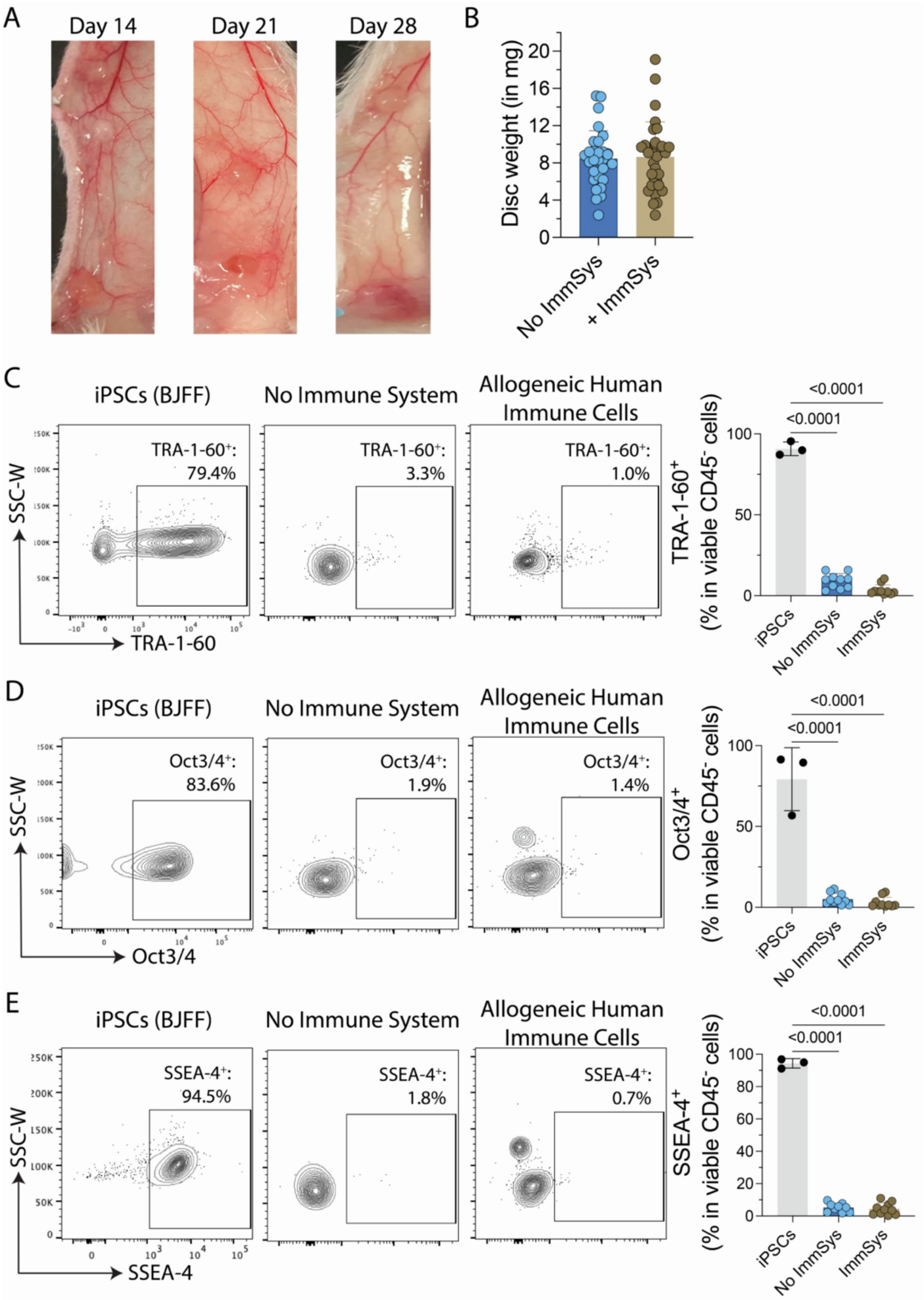

Figure S1. Kidney tissues harvested after transplantation, Related to Figure 2. (**A**) Images of the kidney tissues that were subcutaneously implanted into the NSG mice reconstituted with allogeneic human immune cells, and harvested on days 14, 21, and 28. (**B**) Weights (in mg) of kidney tissues harvested from NSG mice reconstituted with (+ ImmSys) or without (No ImmSys) allogeneic human immune cells. Data represent two pooled experiments with all the time points in the same experimental group (n = 30 - 32 tissues/group). Statistics are performed using an unpaired t-test. Representative contour plots and quantification of (**C**) TRA-1-60$^+$, (**D**) Oct3/4$^+$, and (**E**) SSEA-4$^+$ cells by flow cytometry on kidney discs harvested on day 21 or 28 after the transfer of allogeneic human immune cells. BJFF hiPSCs are used as positive controls. Statistic by one-way ANOVA with Tukey's multiple comparisons test (n = 3 - 10 tissues/group). Data are representative of 2 independent experiments.

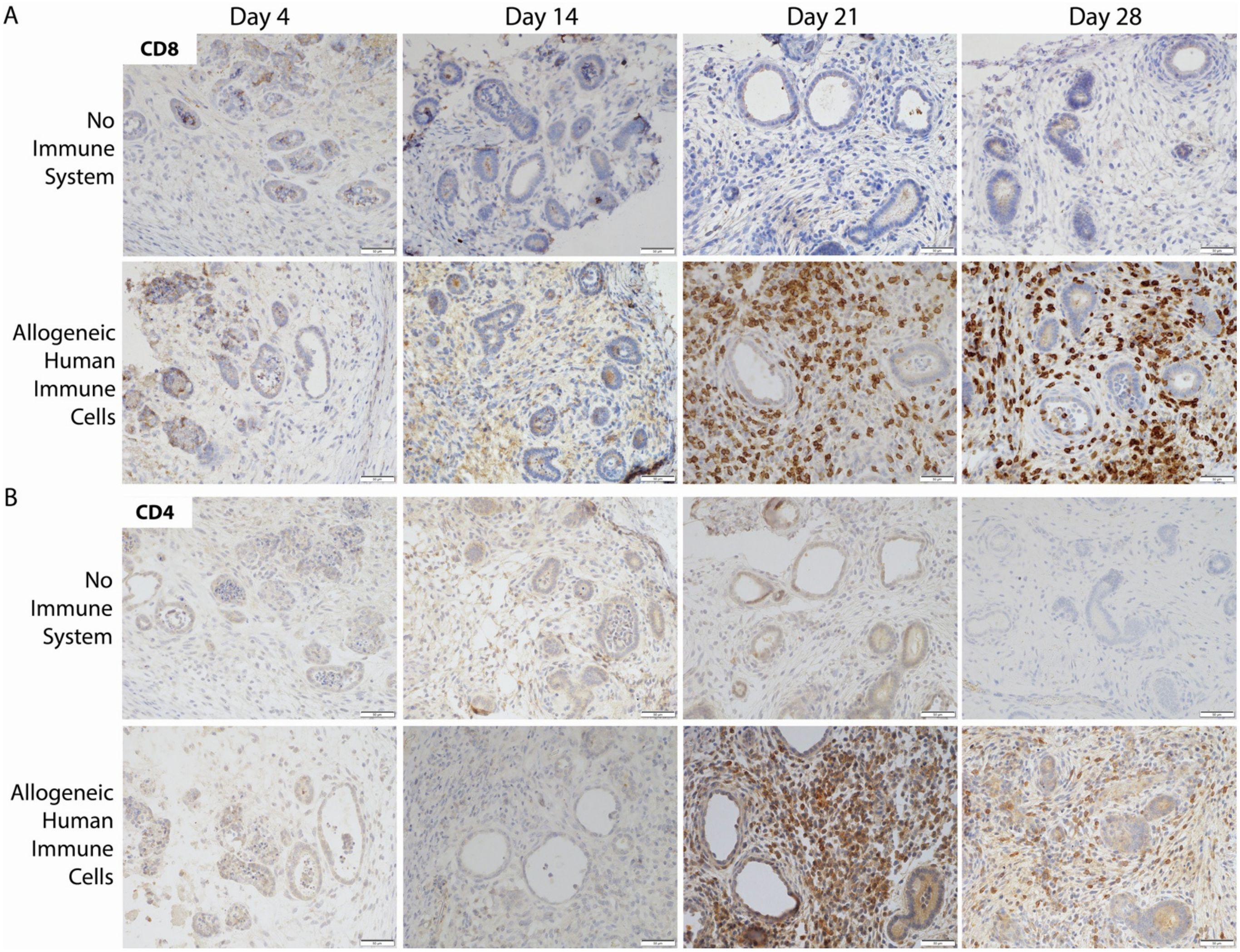

Figure S2. Human kidney disc infiltration by CD8⁺ and CD4⁺ cells, Related to Figure 2. Kidney tissues are transplanted into NSG mice reconstituted with allogeneic human immune cells, harvested and then analyzed on days 4, 14, 21 and 28. Immunostaining showing (**A**) CD8 and (**B**) CD4 expression over time. Data are representative of two independent experiments. Scale bars = 50 μm.

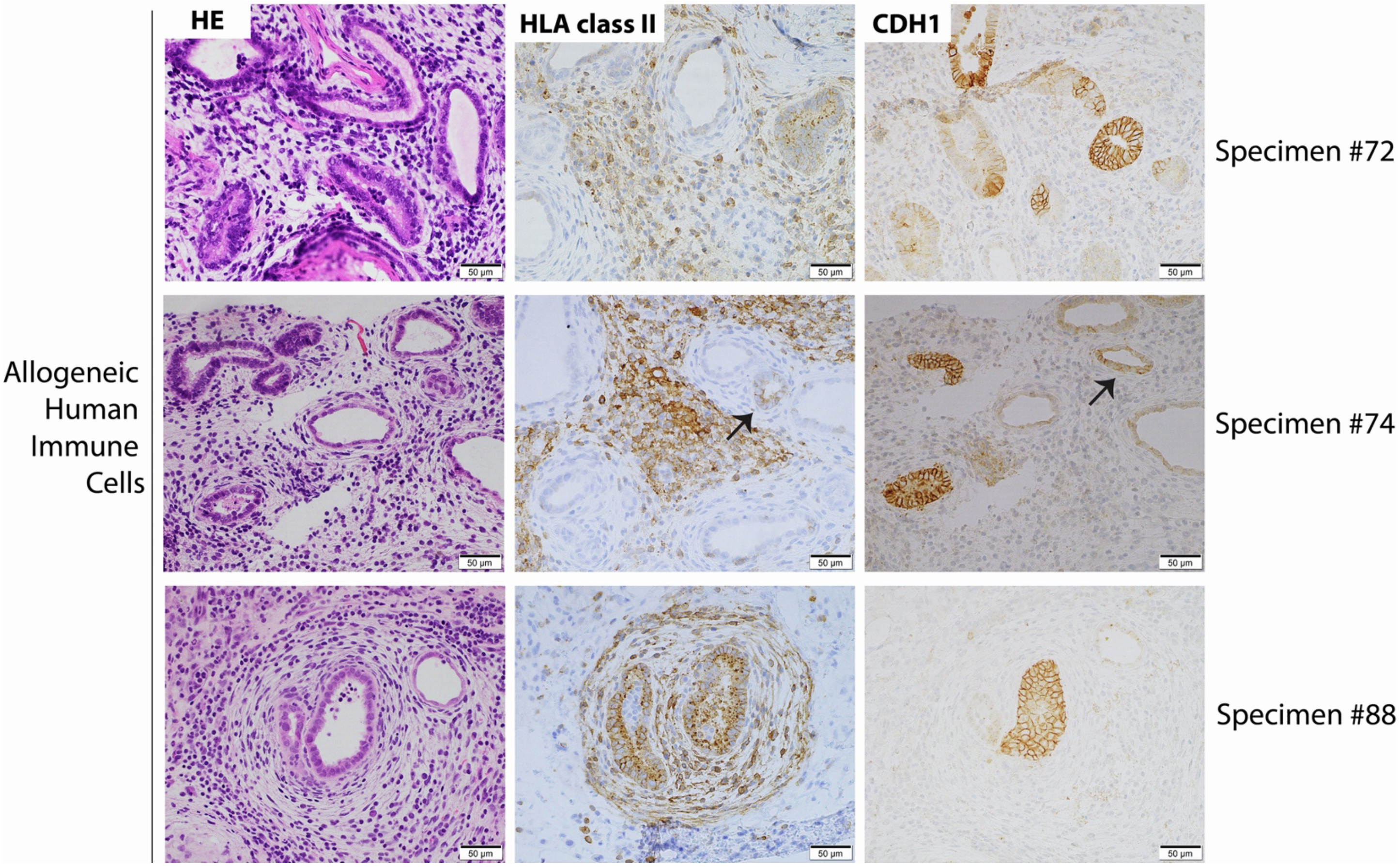

Figure S3. HLA-DR expression by tubule-like structures, Related to Figure 4. Consecutive kidney tissue sections were stained for H&E, HLA-DR and the tubular marker CDH1 on day 21. Scale bars = 50 µm.

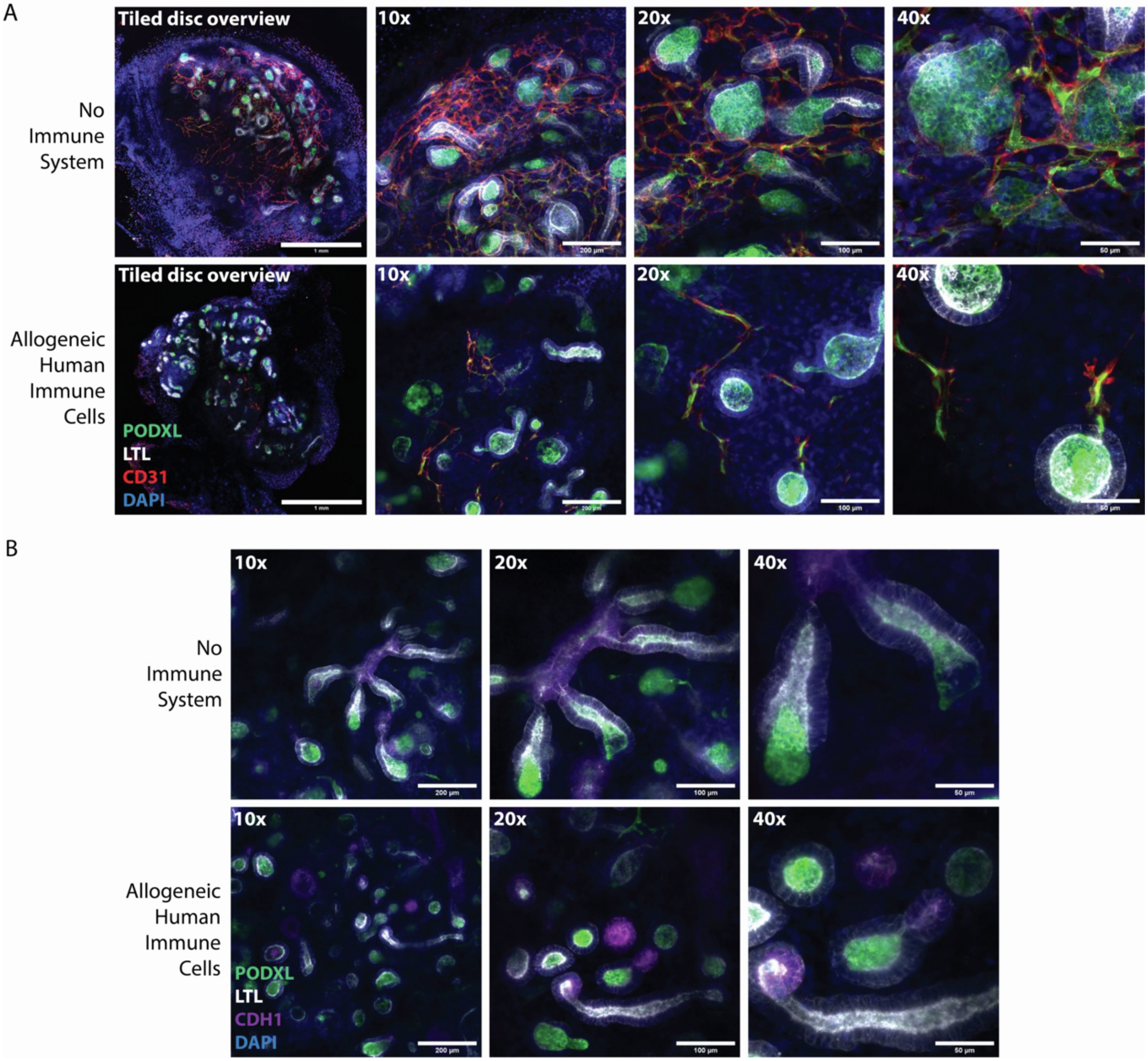

Figure S4. Immunofluorescence imaging of transplanted kidney tissues, Related to Figure 5. (**A**) Confocal images confirming the expression of PODXL (glomeruli, green), LTL (proximal tubules, gray), CD31 (endothelium) and DAPI (nuclei, blue) by tissues harvested on day 21. Scale bars = 1 µm (tiled disc overview), 200 µm (10x), 100 µm (20x), and 50 µm (40x). Data are representative of two independent experiments. (**B**) Confocal images of PODXL (glomeruli, green), LTL (proximal tubules, gray), CDH1 (distal tubules, purple) and DAPI (nuclei, blue) expression by tissues harvested on day 21. Scale bars = 1 µm (tiled disc overview), 200 µm (10x), 100 µm (20x), and 50 µm (40x). Data are representative of two independent experiments.

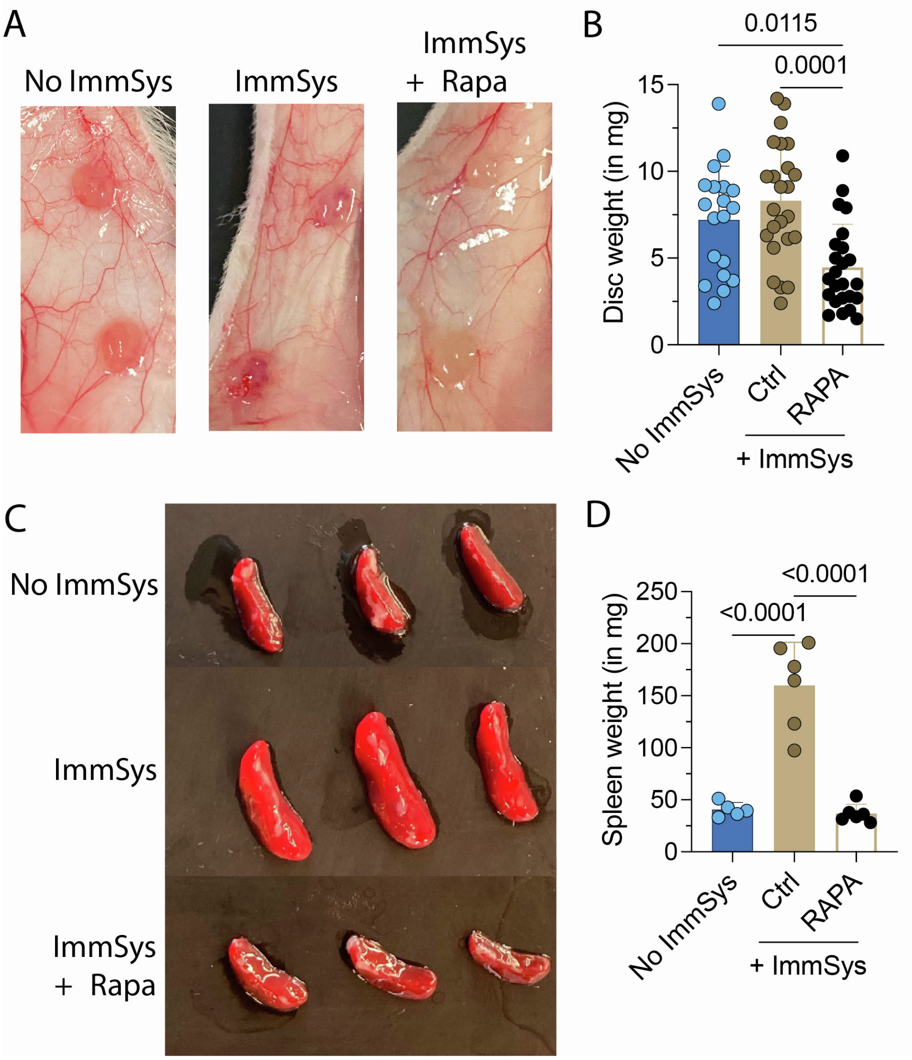

Figure S5. Kidney tissues and spleens harvested after transplantation, Related to Figure 6. Kidney tissues were transplanted into NSG mice reconstituted with (ImmSyst) or without (No ImmSyst) allogeneic human immune cells. One cohort of NSG mice was treated with 200 mg/kg/day of Rapamycin (ImmSyst + Rapa). (**A**) Images and (**B**) weights (in mg) of kidney transplanted tissues harvested on day 21 after the transfer of allogeneic human immune cells. Data are representative of two pooled experiments. Statistic by one-way ANOVA with Tukey post-test (n = 19 - 24 tissues/group). (**C**) Visual aspects and (**d**) weights (in mg) of spleens on day 21 after transferring allogeneic human immune cells. Data are representative of two pooled experiments. Statistic by one-way ANOVA with Tukey post-test (n = 6 spleens/group).

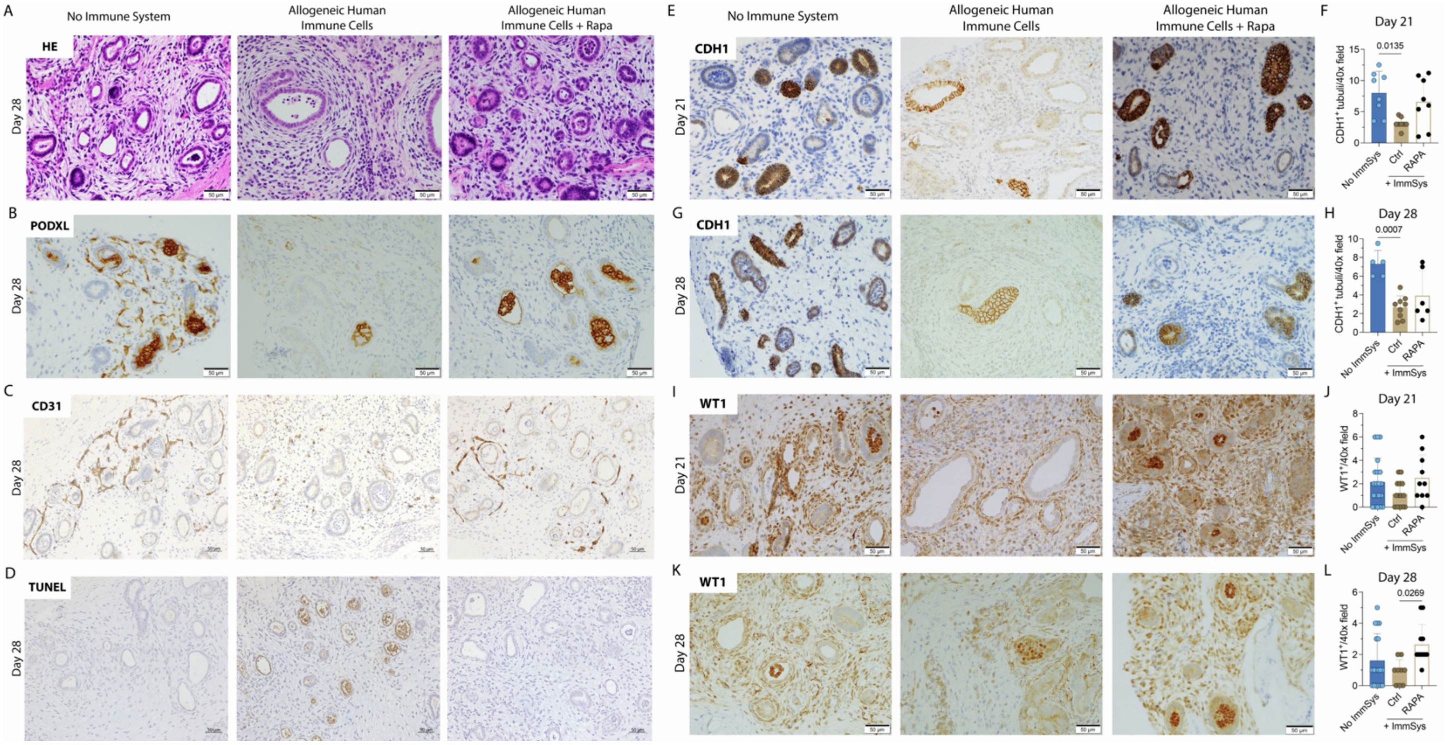

Figure S6. Kidney markers expressed by Rapamycin-treated mice on days 21 and 28, Related to Figure 7. NSG mice are kidney tissue discs implanted, as displayed in Figure 6A. Tissues are harvested and analyzed on days 21 and 28 after the transfer of allogeneic human immune cells. (**A**) Representative H&E on day 28. Representative immunostaining of (**B**) PODXL, (**C**) CD31 and (**D**) TUNEL assay on day 28. Immunostaining and quantification of (**E-H**) CDH1, and (**I-L**) WT1 in the three experimental groups. Scale bars = 50 μm. Statistic by one-way ANOVA with Tukey post-test (n = 5 - 20 tissues/group/time point).

Table S1. HLA typing of iPSCs and immune cells, Related to Figure 2.

	iPSCs	Human Immune Cells
HLA-A	*A*01:01*<br>*A*01:01*	*A*25:01*<br>*A*42:02*
HLA-B	*B*08:01*<br>*B*41:01*	*B*18:01*<br>*B*44:03*
HLA-DRB1	*DRB1*03:01*<br>*DRB1*07:01*	*DRB1*07:01*<br>*DRB1*15:01*

Table S2. Primary antibodies used for immunofluorescence confocal imaging, Related to Methods and Figure S4.

Target	Clone	Concentration	Secondary
PODXL	AF1658	1:300	Anti-Goat
LTL	B-1325	10mg/mL	Biotinylated
CDH1	ab40772	1:300	Anti-Rabbit
PECAM1 (CD31)	ab9498	1:300	Anti-Mouse

Table S4. List of differently expressed genes in transplanted kidney tissues from NSG mice reconstituted with allogeneic human immune cells and treated versus not treated with Rapamycin, Related to Figure 8.

	Log2 fold change	P-value
ALDOC	-1.94	0.0498
HK2	-1.93	0.0994
PDK1	-1.93	0.0501
CXCL14	-1.83	0.0399
PDK1_b	-1.79	0.0696
VEGFA	-1.55	0.0798
ENO1	-1.46	0.0623
ALDOA	-1.15	0.07
PIK3CG	-1.03	0.0771
SLC1A5	-0.849	0.0484
TPI1	-0.837	0.0867
PKM	-0.731	0.0629
BBC3	-0.68	0.0796
PCK2	-0.6	0.0966
HK1	-0.583	0.0998
MYC	-0.524	0.00908
EIF4EBP1	-0.428	0.0566
HDAC4	-0.394	0.0547
SREBF1	-0.359	0.0668
PTPN11	-0.321	0.0399
